# Cold exposure and metabolic health: Therapeutic potential for obesity, diabetes, and beyond

**DOI:** 10.14814/phy2.70838

**Published:** 2026-04-07

**Authors:** Xirong Li, Jiale Dang, Rui Guo, Helen R. Griffiths, Shemin Lu, Dan Gao

**Affiliations:** ^1^ Institute of Molecular and Translational Medicine, School of Basic Medical Sciences Xi'an Jiaotong University Health Science Center Xi'an China; ^2^ Department of Pathology The Second Affiliated Hospital of Xi'an Jiaotong University Xi'an China; ^3^ Swansea University Medical School, Swansea University Swansea UK; ^4^ Key Laboratory of Environment and Genes Related to Diseases (Xi'an Jiaotong University), Ministry of Education Xi'an China; ^5^ Department of Biochemistry and Molecular Biology, School of Basic Medical Sciences Xi'an Jiaotong University Health Science Center Xi'an China; ^6^ Department of Human Anatomy, Histology and Embryology, School of Basic Medical Sciences Xi'an Jiaotong University Health Center Xi'an China

**Keywords:** cancer, cold exposure, diabetes, chronic inflammation, metabolic health, obesity

## Abstract

Cold exposure, an inducer of brown adipose tissue thermogenesis, has been applied to relieve exercise‐induced pain and inflammation, modulate circulation and enhance metabolism. However, its role in managing metabolic diseases and the underlying molecular mechanisms remain inadequately understood. This review provides a comprehensive, updated perspective on the therapeutic potential of cold exposure in obesity, diabetes, inflammation, and cancer. First, we address the physiological effects of cold exposure on adipose tissue thermogenesis and metabolic adaptations. Next, we summarize findings from preclinical and human studies demonstrating that cold exposure reduces serum lipid levels, enhances insulin sensitivity and glucose homeostasis, modulates immune responses, and inhibits tumor growth. Mechanistically, cold sensing is primarily mediated by transient receptor potential channels (TRPM8, TRPA1) in preclinical models, with downstream signaling involving the calcium‐protein kinase A‐uncoupling protein 1 pathway regulating thermogenesis and metabolism. Despite promising potential, cold exposure is linked to stress and cardiovascular risks, especially in vulnerable populations with metabolic disorders or advanced age. The clinical translation is hindered by unstandardized protocols, flawed cooling devices, and incomplete understanding of human brown adipose tissue function. Further research is necessary to elucidate these risks and develop strategies balancing the benefits and harms of cold exposure for clinical translation.

## 1 INTRODUCTION

Cold therapy, or cryotherapy, is an established treatment approach that uses low temperatures to treat various conditions and enhance overall health, such as muscle injury (Racinais et al., 2024), post‐exercise recovery (Allan et al., 2022), and certain types of skin disease (Garcia‐Oreja et al., 2022). Cold therapy typically involves exposing the body or specific body parts to low temperatures, which can be achieved through lower environmental temperature, cold water immersion, cold/ice packs, or cold‐water perfused blankets or vest (Allan et al., 2022). Cold stimulation triggers a series of physiological responses in the body, including vasoconstriction and tissue temperature reduction. These responses offer several potential benefits such as reducing inflammation, pain relief, enhancing muscle recovery, and increasing metabolism (Allan et al., 2022).

It is widely acknowledged that cold exposure induces thermogenesis, which may promote energy expenditure and thus represents a potential approach to treating metabolic disorders, including obesity and diabetes (Ivanova & Blondin, 2021). In humans, cold exposure elicits broad systemic metabolic changes across diverse tissues, including adipose tissue, liver, and muscle (van Beek et al., 2023). Specifically, cold exposure markedly enhances the utilization and metabolism of circulating glucose by skeletal muscle and possibly white adipose tissue (WAT), thereby improving glucose homeostasis (van Beek et al., 2023). The discovery of functional brown adipose tissue (BAT) in adult humans has spurred significant research interest in activating BAT as a potential treatment for human metabolic disease (Cypess et al., 2009; Lee et al., 2011; van Marken Lichtenbelt et al., 2009; Virtanen et al., 2009). Notably, the presence of BAT in humans is more pronounced during cold stimulation (Saito et al., 2009; van Marken Lichtenbelt et al., 2009; Virtanen et al., 2009). However, the metabolic benefits of cold exposure in the context of obesity and its related diseases, along with the underlying molecular mechanisms, remain inadequately understood.

In this review, we aim to provide an up‐to‐date overview of the therapeutic potential of cold exposure for obesity, diabetes, inflammation, and certain types of cancer. We discuss the physiological roles of cold exposure in regulating adipose tissue thermogenesis and metabolic adaptations and summarize findings from preclinical and human research regarding its therapeutic potential. These studies demonstrate that cold exposure lowers circulating lipid levels, improves insulin sensitivity and glucose homeostasis, regulates immune responses, and suppresses tumor growth. Furthermore, we explore the underlying mechanisms of cold‐induced metabolic benefits, as well as the risks of cold exposure in clinical contexts.

## 2 COLD‐INDUCED ADIPOSE TISSUE THERMOGENESIS AND ENERGY ALTERATIONS

Cold‐induced adipose tissue thermogenesis is a vital physiological process for maintaining body temperature and regulating energy metabolism, which has been extensively investigated in both animal models and human studies. Fundamentally, cold induces thermogenesis through two distinct processes, namely shivering and non‐shivering thermogenesis, and these two processes serve as the core basis for understanding the molecular and physiological basis of cold‐induced thermogenesis, which will be discussed in detail in the subsequent sections.

### 2.1 Animal studies on cold‐induced adipose thermogenesis

In mice, non‐shivering thermogenesis is mediated by two distinct forms of thermogenic adipose tissue: classical BAT and beige adipose tissue. Classical BAT originates from My5^+^Pax7^+^ skeletal muscle progenitors during embryonic development and is primarily located in the interscapular area (Seale et al., 2008). Beige adipocytes, by contrast, develop postnatally from Myf5^−^ progenitors, are inducible by cold exposure (Cousin et al., 1992), and reside in inguinal white adipose tissue (iWAT) (Wu et al., 2012). Additionally, lineage tracing studies have identified two unique progenitor populations: Sca‐1^+^Pdgfrα^+^ progenitors give rise to brown adipocytes, while Acta2^+^Myh11^+^ progenitors differentiate into beige adipocytes during cold stimulation (Berry et al., 2016; Lee et al., 2015; Long et al., 2014). In addition to Sca‐1^+^Pdgfrα^+^ progenitors, a recent study demonstrated that Trpv1^+^ progenitors derived from vascular smooth muscle serve as a source of cold‐induced brown adipocytes in mice (Finlin et al., 2018).

In response to cold, the sympathetic nerves release norepinephrine (NE) in mice, which can induce WAT lipolysis and release free fatty acids (FFAs) via the β3‐adrenergic receptor (β3‐AR)/protein kinase A (PKA) pathway (Sakers et al., 2022). FFAs then act as the substrate for activating BAT thermogenesis in a UCP1‐dependent manner (Sakers et al., 2022). In addition to WAT lipolysis, BAT intracellular lipolysis also contributes to cold‐induced BAT thermogenesis in mice, especially during fasting and cold acclimation, as evidenced by the finding that mice with a double‐knockout of adipose triglyceride lipase (ATGL) and hormone‐sensitive lipase (HSL) in BAT exhibit reduced body temperature in the fasted and cold state (Mouisel et al., 2025).

Beyond UCP1‐dependent thermogenesis, several ATP‐dependent futile cycles have been proposed as alternative adipose thermogenesis mechanisms in response to cold stimulation in mice. These include the Sarco/Endoplasmic Reticulum Calcium–ATPase (SERCA)‐mediated calcium cycle (Ikeda et al., 2017), the creatine cycle (Kazak et al., 2015), and the triglyceride (TG)‐FFAs cycle (Anunciado‐Koza et al., 2008). Intriguingly, a recent study demonstrated that the creatine futile cycle operates in parallel with UCP1 and promotes BAT thermogenesis during cold exposure via the SNS/NE/α1‐AR pathway (Rahbani et al., 2024). Furthermore, two research groups independently identified two distinct subpopulations of beige adipocytes in mice, which utilize either a futile cycle or UCP1 for thermogenesis (Vargas‐Castillo et al., 2024; Wang et al., 2024). Moreover, mice subjected to long‐term cold exposure exhibited increased beige fat formation, occurring through de novo beige adipocyte differentiation within iWAT (Cohen & Kajimura, 2021) and the conversion of low‐thermogenic brown adipocytes into high‐thermogenic cells (Song et al., 2020). Additionally, in A/J mice with impaired β3‐AR signaling in BAT, skeletal muscle can compensate for BAT‐mediated nonshivering thermogenesis during cold adaptation and therefore contribute to energy expenditure and resistance to obesity (Bardova et al., 2024). In summary, cold stimulation in mice induces multiple thermogenic mechanisms including both UCP1‐dependent and UCP1‐independent pathways, promoting beige fat formation, and activating skeletal muscle shivering thermogenesis to regulate energy metabolism.

### 2.2 Human studies on cold‐induced adipose thermogenesis

In humans, cold‐induced BAT thermogenesis is not only crucial for maintaining body temperature, particularly in infants, but also confers potential metabolic benefits in adults (Betz & Enerback, 2018). Similar to animal models, human thermogenesis in response to cold relies on two distinct processes: shivering and non‐shivering thermogenesis. During acute cold exposure, muscle shivering serves as the primary heat‐generating mechanism for body temperature maintenance (Castellani & Young, 2016). In contrast, long‐term cold adaptation is associated with attenuated muscle shivering and increased contribution of BAT‐mediated adaptive thermogenesis to heat production (Betz & Enerback, 2018). Notably, signaling through β2‐AR is the primary pathway underlying cold‐activated BAT in humans (Blondin et al., 2020).

BAT abundance and characteristics in humans vary markedly with age. Newborns exhibit relatively high BAT levels (Singh et al., 2021), whereas in adults, BAT is significantly reduced and detectable in only a small subset of individuals (7.5% women and 3.1% men) (Cypess et al., 2009); however, it becomes evident in most adults following cold stimulation (96% men) (van Marken Lichtenbelt et al., 2009). Anatomically, adult human BAT is predominantly localized to paravertebral junctions, cervical regions, and axillary areas (Leitner et al., 2017). Histologically, infant interscapular BAT closely resembles classical murine BAT, characterized by multilocular brown adipocytes (Lidell et al., 2013). In contrast, adult human BAT contains discrete islands of multilocular adipocytes surrounded by unilocular adipocytes, a feature similar to that of murine beige adipose tissue under mild cold conditions (21°C) (Cannon et al., 2020; de Jong et al., 2019). Consistent with these phenotypic differences, gene expression profiling reveals that infant interscapular BAT expresses characteristics of classical brown adipocytes, while adult human BAT displays a transcriptional profile more similar to that of beige adipocytes (Lidell et al., 2013; Shinoda et al., 2015; Wu et al., 2012). However, conflicting evidence exists regarding the nature of human BAT. A recent study using physiologically humanized mice suggests that human supraclavicular BAT may be more similar to murine classical BAT (de Jong et al., 2019; de Jong et al., 2020; Kajimura & Spiegelman, 2020). Notably, these mice were housed at thermoneutrality and fed a high‐fat diet (HFD) for 1 year, which could induce systemic metabolic impairments and thus may not represent an optimal model for mimicking human BAT physiology.

Furthermore, recent advancements in single‐cell or single‐nucleus transcriptomics have uncovered extensive adipocyte heterogeneity in both WAT and BAT. For example, dipeptidyl peptidase‐4 positive (DPP4^+^) stromal vascular fraction (SVF) cells are bona fide multipotent mesenchymal progenitors that give rise to white adipocytes in both murine and human adipose tissue (Nisoli & Cinti, 2024). Importantly, cold exposure has also been shown to induce beiging of subcutaneous WAT in both lean subjects and individuals with obesity, which may provide an additional thermogenic pathway contributing to metabolic benefits (Finlin et al., 2018).

## 3 COLD EXPOSURE‐INDUCED METABOLIC ALTERATIONS

Cold exposure profoundly regulates whole‐body metabolism in a species‐specific manner. Given the distinct responses observed between rodents and humans, we separately outline cold‐induced metabolic alterations in mice and humans to clearly differentiate preclinical data from clinical observations.

### 3.1 Cold‐induced metabolic alterations in animal models

**FIGURE 1:**
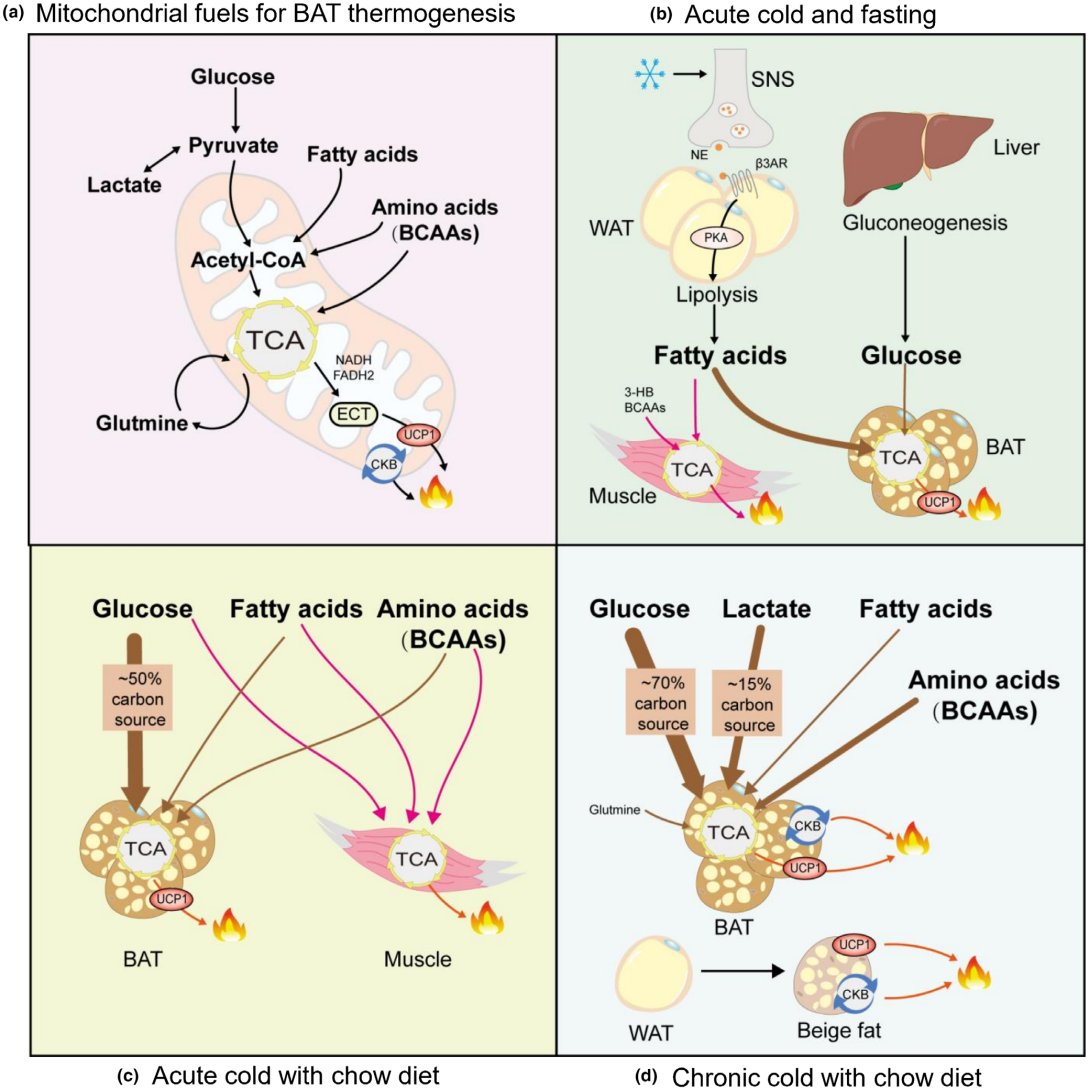
Cold‐induced adipose adaptive thermogenesis and metabolic changes. (a) Mitochondrial metabolic pathways in thermogenesis. Mitochondrial metabolic fuels enter the tricarboxylic acid (TCA) cycle, contributing to uncoupling protein 1 (UCP1)‐dependent thermogenesis. Concurrently, the creatine kinase B (CKB)‐mediated creatine futile cycle operates in parallel with UCP1 to enhance brown adipose tissue (BAT) thermogenesis. (b) Substrate mobilization under acute cold and fasting conditions. During acute cold exposure and fasting, fatty acids are released from triglyceride stores in white adipose tissue (WAT), while glucose is generated via liver gluconeogenesis to support the TCA cycle in BAT for thermogenesis. Fatty acids serve as the primary fuel for heat production in BAT. In muscle, branched‐chain amino acids (BCAAs) and liver‐derived 3‐hydroxybutyrate (3HB) act as key energy sources for the thermogenesis process. (c) Fuel preference during acute cold exposure with chow diet. Under acute cold exposure and fed a chow diet, BAT primarily utilizes glucose thermogenesis. (d) Metabolic flexibility during chronic cold exposure. During chronic cold exposure on a chow diet, BAT exhibits a significant increase in the uptake of diverse circulating fuel metabolites, including glucose, lactate, 3HB, long‐chain fatty acids, and most amino acids (e.g., BCAAs). Glucose and lactate serve as the dominant fuels for BAT thermogenesis, accounting for approximately 85% of the carbon source utilized. Additionally, the glutamine synthesis and catabolism cycle may function as an alternative futile cycle to support BAT thermogenesis. BAT, brown adipose tissue; BCAAs, branched‐chain amino acids; CKB, creatine kinase B; TCA, tricarboxylic acid; UCP‐1, uncoupling protein 1; WAT, white adipose tissue.

In mouse models, multiple fuel sources including free fatty acids, glucose, lactate, amino acids, and glutamine are utilized by BAT mitochondria for thermogenesis during cold exposure (Figure 1a). Using arteriovenous sampling and stable isotope tracing, metabolic fluxes across tissues have been comprehensively quantified in cold‐exposed mice (Bornstein et al., 2023; Park et al., 2023). Specifically, in mice subjected to acute cold exposure and fasting (Figure 1b), fatty acids released from TG stores in WAT serve as the primary energy source for BAT thermogenesis (Bornstein et al., 2023). Moreover, acute cold exposure in fasted mice promotes liver gluconeogenesis to support the tricarboxylic acid (TCA) cycle in BAT (Bornstein et al., 2023), while branched‐chain amino acids (BCAAs) and liver‐derived 3‐hydroxybutyrate (3HB) fuel muscle thermogenesis (Bornstein et al., 2023; Xu et al., 2019). Conversely, in acutely cold‐exposed mice fed a chow diet, BAT predominantly uses glucose for thermogenesis (Bornstein et al., 2023; Park et al., 2023) (Figure 1c). These preclinical findings in mice suggest that cold exposure reshapes whole‐body metabolism via tissue‐specific fuel selection, orchestrating complex inter‐tissue crosstalk.

Chronic cold exposure further alters metabolism in preclinical mouse models. In ad libitum chow‐fed mice, chronic cold exposure increases BAT uptake of multiple circulating metabolites, including glucose, lactate, 3HB, long‐chain fatty acids, and most amino acids (including BCAAs) (Figure 1d) (Park et al., 2023). Among these, fatty acids are the preferential fuels for BAT thermogenesis during long‐term cold exposure in mice (Park et al., 2025). Chronic cold exposure also induces glutamine synthesis and catabolism in mouse BAT, a metabolic pattern that potentially supports BAT thermogenic function (Park et al., 2023). Overall, in mice, BAT's enhanced uptake of circulating metabolites during prolonged cold exposure sustains its role in body temperature maintenance. This unique property of mouse BAT confers metabolic benefits in preclinical settings and implies therapeutic potential for metabolic diseases.

### 3.2 Cold‐induced metabolic alterations in humans

In humans, acute cold stimulation appears to significantly modulate glucose and lipid metabolism (Table 1). In healthy human subjects, acute mild‐cold exposure (ambient temperature of 17 ± 1°C for ~2 h) increases BAT glucose (Orava et al., 2011; Virtanen et al., 2009) and FFAs uptake (Ouellet et al., 2012), improves insulin sensitivity by approximately 20% (Iwen et al., 2017), but does not alter pancreatic insulin secretion (Iwen et al., 2017). Beyond BAT, skeletal muscle contributes to glucose uptake during acute cold exposure accompanied by shivering in humans (Blondin et al., 2015). Consistent with this, healthy human males exposed to cold conditions with shivering (7.5°C for 24 h) display reduced total carbohydrate oxidation and elevated lipid oxidation (Haman et al., 2016). However, acute cold exposure (10°C for 1 h, with shivering) followed by 90 min of rest does not affect glucose tolerance in healthy subjects (Sellers et al., 2021), suggesting that cold‐induced changes in glucose metabolism may be transient in humans.

**TABLE 1:** Cold exposure and human studies.

References	Participants	Achieved CE temperature	CE modality	CE duration	Outcome	Detection of BAT activity
*Healthy subjects*						
van Marken Lichtenbelt et al. (2009)	24 males (10 lean and 14 ow/ob)	16°C	Air	2 h	BAT activity↑	PET‐CT
Virtanen et al. (2009)	5 subjects	5°C–9°C	One‐foot intermittent in ice water	2 h (5 min each time)	BAT glucose uptake↑ (15‐fold)	PET‐CT
Saito et al. (2009)	56 subjects (25F/31M)	19°C	Air and intermittent ice‐cooled footrest treatment	2 h	BAT glucose uptake↑	PET‐CT
Orava et al. (2011)	27 subjects (20F/7M)	17 ± 1°C	Air	2 h	BAT glucose uptake↑ (12‐fold)	PET‐CT
Iwen et al. (2017)	15 males	18°C	Air	2 h	Peripheral glucose uptake and insulin sensitivity↑ (20%)	PET‐CT
Ouellet et al. (2012)	6 males	18°C	Liquid‐cooled suit	3 h	EE↑ (1.8‐fold) BAT NEFA uptake↑	PET‐CT
Jurado‐Fasoli et al. (2024)	64 subjects (47F/17M)	19.5°C (air)	Air and water cooling vest	2 h	Omega‐6 oxylipins ↑ (47%) Omega‐3 oxylipins↑ (77%)	PET‐CT
Yoneshiro et al. (2013)	51 males (27 BAT^+^; 24 BAT^−^)	17°C	Air	6 weeks (2 h each day)	BAT^+^ group: BAT activity↑ (58%) EE ↑ (~20%) Body fat mass↓ (5%)	PET‐CT
Blondin et al. (2017)	8 males	18°C	Liquid‐cooled suit	4 weeks (2 h each day)	BAT oxidative capacity ↑ (2.6‐fold)	PET‐CT
Acosta et al. (2018)	11 males	19.4 ± 0.1°C (air)	Air and water cooling vest	2 h	REE ↑ (~16%)	——
Sellers et al. (2021)	16 males	10°C	Water‐perfused suit	1 h	EE ↑ (~2‐fold)	——
Horing et al. (2025)	171 subjects (93F/78M)	2°C above shivering temperature	Water‐perfused blankets	2 h	Almost all serum lipids levels ↑ including TG ↑ FFA↑	——
Hoeke et al. (2017)	24 males	3°C above shivering temperature	Water‐perfused cooling mattresses	2 h	Serum TG ↑ HDL↑ FFA↑	——
Eimonte et al. (2022)	17 males	14°C	Whole‐body cold‐water immersion	170 min (intermittent 20 min/session for 6 sessions)	Serum TG ↑ HDL↑	——
*Subjects with obesity*						
Mengel et al. (2022)	Lean 59F/58M ow 13F/14M; ob 15F/11M	2°C above shivering temperature	Water perfused blankets	2 h	REE↑ in lean subjects (6%) REE ↔ in subjects with ow/ob	——
Kulterer et al. (2022)	Lean 18F/13M ob 38F/26M	2°C above shivering temperature	Water‐perfused cooling vest	3 h	BAT^+^ group: EE↑ (~7%) in both lean and subjects with obesity	PET‐CT
McInnis et al. (2025)	29F/18M	10°C	Liquid‐cooled suit	1.5 h	EE↑(18%) EI↑(10%)	——
Chondronikola et al. (2014)	12 males (7 BAT^+^; 5 BAT^−^)	19°C	Liquid‐cooled vest and blanket	5–8 h	BAT^+^ group: REE↑ (15%) Insulin sensitivity↑	PET‐CT
Hanssen et al. (2016)	10 males with ow/ob	14°C–15°C	Air	10 days (1 h on day 1, 4 h on day 2, 6 h on day 3–10)	BAT activity↑ EE↑ (14%) BAT and SM glucose uptake↑	PET‐CT
Brychta et al. (2019)	21 males (12 lean; 9 ob)	16°C	Air	10 days	Lean: EE↑↑ (17%) Ob: EE↑ (6%)	PET‐CT
Sellers et al. (2024)	15 subjects with ow/ob (4F/11M)	10°C	Water perfused suit	10 days (1 h of shivering per day)	EE↔ Fasting glucose↓ FFAs, TG↓	——
*Subjects with T2DM*						
Mooventhan et al. (2020)	8 subjects with T2DM (3F/5M)	15°C–16°C	Cold hip bath	20 min	Random blood glucose↓ (18%)	——
Hanssen et al. (2015)	8 males with ow + T2DM	14°C–15°C	Air	10 days (2 h on day 1, 4 h on day 2, 6 h on day 3–10)	EE↑ (5%) Insulin sensitivity↑ 43% SM GLUT4 translocation↑	PET‐CT
Remie et al. (2021)	9 subjects with ob + T2DM (4F/5M)	16°C–17°C	Air	10 days (2 h on Day 1, 4 h on Day 2, 6 h on Day 3–10)	EE↑	——
*Subjects with cancer*						
Seki et al. (2022)	A patient with Hodgkin's lymphoma	22°C	Air	7 days	BAT glucose uptake↑ Tumor glucose uptake↓	PET‐CT

Abbreviations: CE, cold exposure; DFA, dietary fatty acids; F, female; FFAs: free fatty acids; GLTU4, glucose transporter 4; M, male; ob, obese (BMI ≥ 30 kg/m^2^); ow, overweight (BMI 25.0–29.9 kg/m^2^); PET‐CT, positron emission tomography–computed tomography; REE, resting energy expenditure; SM, skeletal muscle; T2DM, type 2 diabetes mellitus; TG, triglycerides.

Acute cold exposure also alters nearly all serum lipids levels in healthy humans (Horing et al., 2025). For example, 2 h of cooling via a cold‐water perfused mattress or 170 min of intermittent cold‐water bathing (both without shivering) increases serum FFAs, TGs, and HDL in humans (Eimonte et al., 2022; Hoeke et al., 2017; Horing et al., 2025). In contrast, 10 min of cold‐water immersion has no effect on serum TC, TGs, or LDL levels (Eimonte et al., 2022). These divergent results in human studies may be due to increased WAT lipolysis and hepatic VLDL‐TG production, driven by enhanced systemic sympathetic activity (Geerling et al., 2014).

Additionally, acute cold exposure reshapes the circulating fatty acid profile (Straat et al., 2022). Specifically, levels of long‐chain monounsaturated fatty acids (C22:0) and very long‐chain saturated fatty acids (C20:1n9 and C24:1n9) decrease in human subjects (Iwen et al., 2017). A recent human trial further reported that 2 h of cold exposure raises plasma signaling lipids (omega‐6 oxylipins by 47% and omega‐3 oxylipins by 77%) in young adults (Jurado‐Fasoli et al., 2024). These changes correlate with a healthier cardiometabolic profile, including lower adiposity, improved glucose homeostasis, more favorable lipid levels, and better liver function markers (Jurado‐Fasoli et al., 2024). Importantly, this association is independent of BAT activation, as only 5% of the total measured lipid species show a weak negative correlation with BAT volume (Jurado‐Fasoli et al., 2024). This implies that non‐BAT mechanisms contribute to cold‐induced improvements in the cardiometabolic lipid profile in humans.

Furthermore, a 4‐week human study of mild‐cold exposure (18°C, 2 h/day) demonstrated that this intervention increases BAT oxidative capacity by 2.6‐fold but does not enhance dietary fatty acids (DFA) uptake or systemic fatty acid clearance (Blondin et al., 2017). This finding indicates that BAT plays a minimal role in DFA clearance in humans compared with other organs such as the heart, liver, skeletal muscles, or WAT. Given the relatively small volume of BAT in humans, it remains debatable whether BAT activation alone can serve as the primary driver of substantial metabolic improvement (Gupta, 2023).

## 4 MOLECULAR MECHANISMS OF COLD‐INDUCED METABOLIC CHANGES

Thermal sensation, encompassing cold and heat perception, is a fundamental component of somatic sensation. Among the receptors mediating thermal sensation, the transient receptor potential (TRP) channel family is critical. These protein‐based transducers detect and relay environmental stimuli, including temperature, pain, tactile pressure, and osmotic pressure. Two key TRP channels involved in cold sensation, TRPM8 and TRPA1, are analyzed in detail below, with clear distinctions between preclinical and human studies.

### 4.1 Role TRPM8 in cold sensation and metabolic regulation

In preclinical mouse models, TRPM8 serves as the primary transducer for non‐noxious cold sensation in primary afferent neurons (Bautista et al., 2007). As a calcium ion channel, it exhibits high sensitivity to non‐noxious cold temperatures (below 25 °C) and cooling agents such as menthol and icilin (Bautista et al., 2007; Dhaka et al., 2007). TRPM8 expression is widely distributed across various tissues in rodents, particularly in those critical for cold perception, including skin, dental pulp, nasal mucosa, and respiratory airways (Liu et al., 2020). Notably, studies using rodent models have shown that TRPM8 transcripts are expressed in small‐diameter neurons of the trigeminal ganglia and dorsal root ganglia (DRG), which contain subpopulations of C and Aδ afferent nerve fibers (Kobayashi et al., 2005). Functional studies using HEK293T cells have confirmed that the C‐terminal domain of rat TRPM8 is responsible for mediating its temperature sensitivity (Brauchi et al., 2006). Furthermore, experiments in TRPM8‐overexpressing CHO cells demonstrated that a temperature decrease from 25°C to 15°C enhances intracellular Ca^2+^ influx (Peier et al., 2002); the resultant alterations in transmembrane calcium concentration generate bioelectrical signals relayed to the central nervous system, ultimately mediating cold perception (Peier et al., 2002).

Consistent with rodent data, TRPM8 is also widely expressed in human tissues, with high abundance in cold perception‐related sites such as the skin, dental pulp, nasal mucosa, and respiratory airways (Liu et al., 2020). Studies have confirmed TRPM8 expression in human dorsal root ganglia (DRG) neurons (Nguyen et al., 2021). However, the detailed molecular mechanism underlying its temperature sensitivity (e.g., the role of the C‐terminal domain) has not been fully validated in human‐derived cells, leaving gaps in our understanding of its functional role in human cold perception.

All evidence linking TRPM8 activation to metabolic regulation comes from rodent models, with no supporting human clinical data. In these preclinical settings, TRPM8 activation mimics cold stimulation and exerts metabolic benefits, showing promise for improving outcomes in obese and type 2 diabetic mice (Sanders et al., 2021). For example, feeding mice a diet supplemented with menthol (a TRPM8 agonist) induces thermogenesis and protects against high‐fat diet (HFD)‐induced obesity and glucose intolerance via the TRPM8‐Ca^2+^‐PKA‐UCP1 pathway (Ma et al., 2012). Notably, TRPM8 is also expressed in rodent brown adipocytes, and menthol treatment upregulates UCP1 expression in these cells (Ma et al., 2012), suggesting an alternative pathway for cold‐induced thermogenesis in BAT. Acute and chronic topical menthol treatment further activates BAT in mice, increasing UCP1 expression and thermogenesis (Sankina et al., 2024); these effects persist even in mice housed at thermoneutrality (McKie, Medak, et al., 2022). Global *Trpm8* knockout mice develop age‐related obesity and glucose intolerance at room temperature, a phenotype partially driven by daytime hyperphagia and reduced fat oxidation (Reimundez et al., 2018). Critical insights from conditional knockout models show that neuron‐specific *Trpm8* knockout mice develop obesity on a chow diet, whereas peripheral sensory neuron‐specific *Trpm8* knockout mice do not (Liskiewicz et al., 2023), indicating that the anti‐obesity effect of TRPM8 is likely mediated by central nervous system stimulation rather than peripheral sensory neurons. Similarly, subcutaneous injection of icilin, another TRPM8 agonist, elicits positive effects in HFD‐fed mice, such as increased energy expenditure and reduced body weight (Clemmensen et al., 2018); unlike menthol, however, icilin exerts no direct cell‐autonomous effects on cultured brown adipocytes (Liskiewicz et al., 2023).

### 4.2 Role TRPA1 in cold sensation and metabolic regulation

In preclinical mouse models, TRPA1, a TRP family member also known as ankyrin‐like protein with transmembrane domains protein 1 (ANKTM1), serves as a nociceptive receptor in sensory neurons, specifically detecting noxious cold stimuli (temperatures below 18°C) (Story et al., 2003). Notably, TRPA1 is co‐expressed with TRPV1/VR1, the well‐characterized capsaicin/heat receptor, but not with TRPM8 (Story et al., 2003), a distinct profile that supports the limited role of TRPM8 in noxious cold sensing. However, a recent study reported conflicting findings. A substantial proportion of TRPM8^+^ cold‐sensing nerve fibers in the cornea co‐express TRPV1 (Li et al., 2019). This co‐expression enhances neuronal excitability and ocular cold nociception (Li et al., 2019), indicating that TRP receptor co‐expression patterns are tissue‐specific rather than universal. In contrast to the robust preclinical data, research on human TRPA1 in cold perception is relatively scarce. Existing evidence only preliminarily confirms TRPA1's basic expression in human sensory neurons, with insufficient clinical data to validate its specific response to noxious cold (below 18°C) or its co‐expression patterns with TRPV1/TRPM8. Whether the expression characteristics and functional mechanisms of human TRPA1 align with those in animal models remains unconfirmed.

TRPA1 activation also confers metabolic benefits in rodent models. For instance, treating mouse 3T3‐L1 adipocytes with cinnamaldehyde (CIN), a TRPA1 agonist, reduces lipid accumulation and downregulates key adipogenic transcription factors (PPARγ, C/EBP‐α, and C/EBP‐β) (Hoi et al., 2020). In contrast, the TRPA1 inhibitor AP‐18 enhances TG synthesis in adipocytes (Hoi et al., 2020). In vivo, a single oral gavage of CIN in mice activates TRPA1 in gastric epithelial cells and inhibits the production of ghrelin, the hunger hormone (Camacho et al., 2015). This reduces food intake and slows gastric emptying, ultimately alleviating obesity and improving glucose tolerance (Camacho et al., 2015). CIN also induces thermogenesis in adipocytes derived from mouse primary adipose stem cells (Jiang et al., 2017), thereby reducing HFD‐induced obesity in mice (Zuo et al., 2017). However, the exact role of TRPA1 in thermogenesis remains uncertain and debate persists over whether its activation mimics cold stimulation as TRPM8 does. Despite TRPM8's established role as a key cold‐sensing receptor, the contribution of TRPA1 activation to cold mimicry and metabolic regulation is not fully understood, necessitating further research to clarify its similarities and differences with TRPM8.

Research on TRPA1 and human metabolism is limited to in vitro experiments using human‐derived cells, with no in vivo clinical data available. CIN has been shown to induce thermogenesis in adipocytes derived from human primary adipose stem cells (Jiang et al., 2017), but the physiological relevance and clinical translational value of this effect in humans remain invalidated. The specific role and molecular mechanism of TRPA1 in human metabolic regulation thus require further confirmation through large‐scale clinical studies.

In summary, TRPM8 and TRPA1, two key cold‐sensing TRP channels, are closely linked to energy metabolism, and their activation holds potential for treating obesity and other metabolic diseases. Notably, most current evidence for their regulatory roles comes from preclinical animal models, with human research remaining scarce and limited to in vitro studies. Elucidating how these neural cold receptors regulate metabolic processes will require integrating more human clinical data with preclinical findings, providing a robust basis for developing novel therapeutic strategies for metabolic disorders.

## 5 COLD EXPOSURE AND OBESITY

Cold exposure, a potent inducer of BAT thermogenesis and energy expenditure, has attracted considerable research interest for its implications in body weight regulation and obesity treatment (Table S1). Below is a detailed analysis of relevant findings, with clear distinctions between preclinical (animal) and human study results.

### 5.1 Preclinical studies

Preclinical studies on cold exposure and obesity primarily use rodent models, which provide valuable insights into the underlying mechanisms of cold‐induced metabolic regulation due to their well‐characterized adipose tissue physiology and genetic tractability. These studies focus on dissecting the effects of different cold exposure paradigms, metabolic outcomes, and interactions with therapeutic agents, laying the groundwork for understanding translational potential to humans.

#### 5.1.1 Effects of sustained and intermittent cold exposure on body weight

In rodent models, sustained cold exposure exerts consistent anti‐obesity effects regardless of dietary regimen. When fed either a chow or high‐fat diet (HFD), rodents subjected to continuous prolonged cold exposure (4°C for over 1 week) exhibit increased energy expenditure (Vaanholt et al., 2009) and reduced body weight, even with elevated food intake (Harri et al., 1984; Kibler & Johnson, 1961; Park et al., 2023; Vallerand et al., 1986; Yahata & Kuroshima, 1989). Prolonged mild cold exposure also elicits beneficial effects. For example, exposure to 12°C–17°C for 4 weeks (Zietak et al., 2016) or 22°C for 5 days (van der Stelt et al., 2017), reduces fat mass and adiposity in HFD‐fed obese mice. Consistently, sustained cold exposure is accompanied by increased BAT mass (Harri et al., 1984; Vallerand et al., 1986; Wang, Che, et al., 2015; Yahata & Kuroshima, 1989) and activity (Paulus et al., 2020), indicating that BAT activation serves as a key mediator of cold‐induced body weight changes. This is further supported by experiments showing that housing mice under thermoneutral conditions to inactivate BAT diminishes or reverses the weight‐loss effects of cold exposure (Aldiss et al., 2022; McKie, Shamshoum, et al., 2022), confirming the critical role of BAT in this process.

In contrast, studies on intermittent cold stimulation have yielded highly divergent results owing to variations in experimental designs. Most studies failed to detect changes in body weight or fat mass, or only observed minor reductions in fat mass (da Silva et al., 2020; Huo et al., 2022; Raun et al., 2024; Ravussin et al., 2014; Wang, Liu, et al., 2015). Surprisingly, some reports even suggest that intermittent cold stimulation may promote weight gain. The underlying mechanisms for this discrepancy remain unclear, potentially involving hyperphagia (McKie, Shamshoum, et al., 2022) or cold‐induced activation of fat synthesis in the liver and white adipose tissue (WAT) (Yoo et al., 2014). In addition, the effects of cold stimulation vary across animal models. For example, 2 weeks of continuous cold exposure (3°C–35°C) did not alter body weight in Swiss mice (Zhao et al., 2022), an effect attributed to compensatory changes where reduced adipose tissue mass was counterbalanced by weight gain in other organs such as liver and intestines. Targeted local cold stimulation (e.g., restricted to mice's feet) also fails to improve body weight or fat mass (Zhu et al., 2018). Collectively, sustained cold exposure reliably induces weight loss in rodents via BAT activation, whereas intermittent cold stimulation and studies using non‐generalizable animal models yield inconsistent outcomes.

#### 5.1.2 Effects of cold exposure on glucose and lipid metabolism in obesity

Beyond energy expenditure, cold exposure improves glucose tolerance, insulin sensitivity, and serum lipids profiles in obese rodent models. Prolonged cold exposure (4–10 weeks at 4°C) in HFD‐fed obese Wistar rats or mice enhances glucose tolerance and insulin sensitivity (Vallerand et al., 1986; van der Stelt et al., 2017; Zietak et al., 2016), while reducing serum TG, TC, and low‐density lipoprotein (LDL) levels (He et al., 2021; Pernes et al., 2021). However, inconsistencies exist, as 10 days of cold exposure at 4°C fail to improve insulin resistance in obese *fa/fa* rats and instead increase serum TG levels (Bobbioni‐Harsch et al., 1994), likely due to phenotypic differences between obese animal models.

**FIGURE 2:**
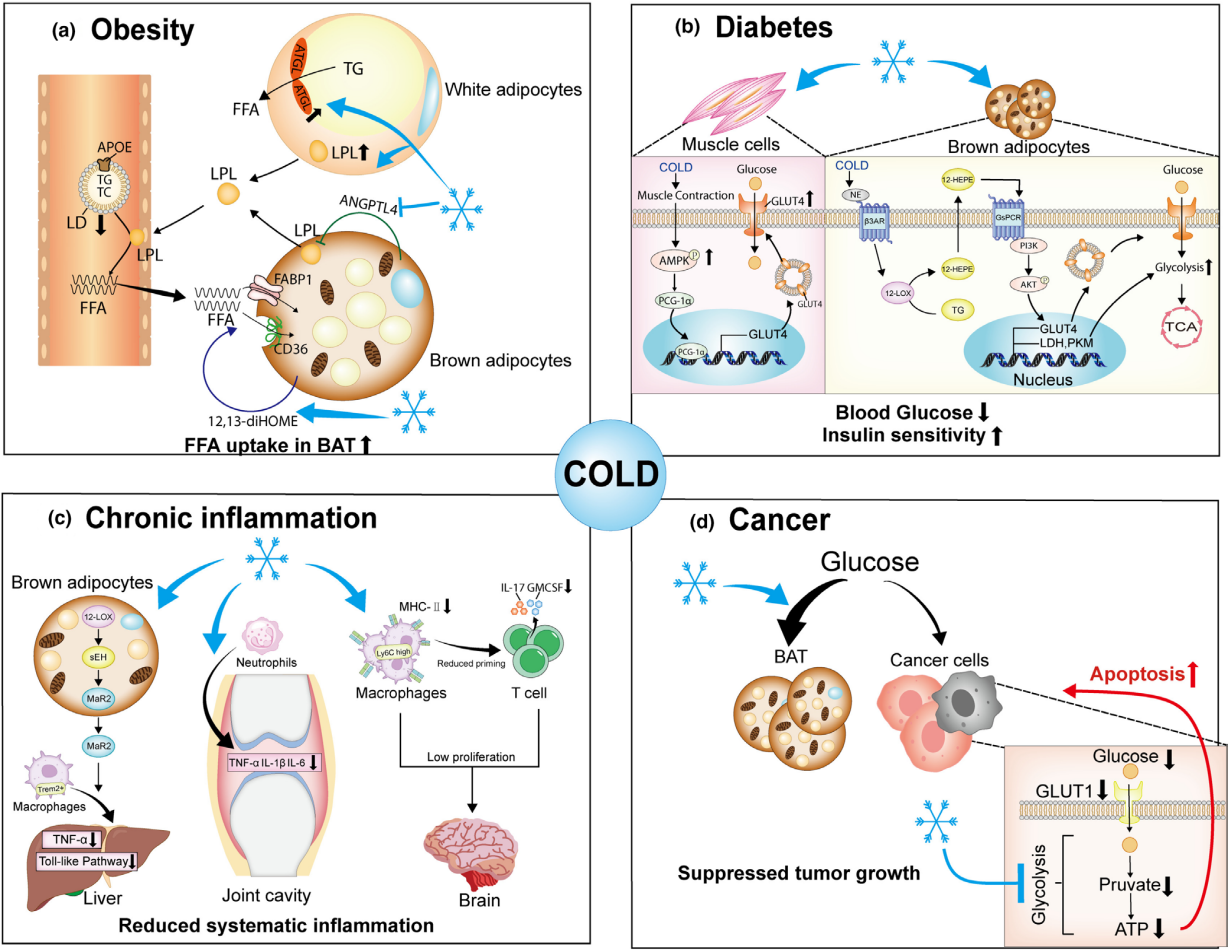
Cold stimulation in the context of obesity, diabetes, chronic inflammation, and cancer. (a) In obesity, cold stimulation promotes lipolysis by enhancing adipose triglyceride lipase (ATGL) activity and upregulates lipoprotein lipase (LPL) expression in white adipocytes. Through the action of adipokines/lipokines, it also promotes the uptake of free fatty acids (FFAs) by brown adipocytes. Collectively, these mechanisms reduce circulating lipid levels in individuals with obesity. (b) In diabetes, cold stimulation activates the β3‐adrenergic receptor (β3‐AR)/12‐lipoxygenase (12‐LOX)/12‐hydroxyeicosapentaenoic acid (12‐HEPE)/Gs/phosphatidylinositide 3‐kinase (PI3K)/AKT pathway in brown adipocytes. This activation promotes glucose transporter 4 (GLUT4) translocation, thereby enhancing glycolysis and the tricarboxylic acid (TCA) cycle activity. Concurrently, cold exposure activates the AMP‐activated protein kinase (AMPK)/peroxisome proliferator‐activated receptor gamma coactivator 1‐alpha (PGC1‐α) pathway in skeletal muscle cells, facilitating GLUT4 translocation to the cell membrane. These actions lower blood glucose levels and improve glucose tolerance in individuals with type 2 diabetes (T2DM). (c) During obesity‐associated chronic inflammation, cold stimulation activates the 12‐LOX‐soluble epoxide hydrolase (sEH)‐maresin 2 (MaR2) pathway in brown adipocytes, triggering the release of MaR2—a bioactive lipid that promotes inflammation resolution. This lipid enhances the migration of Trem2^+^ anti‐inflammatory macrophages to the liver, thereby reducing hepatic inflammation. Moreover, cold stimulation reduces chemotaxis of inflammatory cells and production of inflammatory factors, alleviating the chronic inflammation in joints and brain. (d) In cancer, cold stimulation induces systemic glucose redistribution: Increased glucose uptake by brown adipose tissue (BAT) and reduced glucose supply to cancer cells, thereby inducing their apoptosis. 12‐HEPE, 12‐hydroxy‐eicosapentaenoic acid; 12‐LOX, 12‐lipoxygenase; AMPK, Adenosine 5′‐monophosphate‐activated protein kinase; ATGL, adipose triglyceride lipase; BAT, brown adipose tissue; FFAs, free fatty acids; GLUT4, glucose transporter 4; LPL, lipoprotein lipase; MaR2, maresin 2; PGC‐1α, PPARγ coactivator‐1α; PI3‐K, phosphatidylinositide 3‐kinase; sEH, soluble epoxide hydrolase; T2DM, type 2 diabetes; TCA, trichloroacetic acid; β3‐AR, β3‐adrenergic receptor.

Mechanistically, cold exposure modulates lipid metabolism through multiple pathways in rodents. BAT from mice exhibits greater mass‐specific TG uptake efficiency than skeletal muscle or WAT, contributing to circulating TG clearance (Bartelt et al., 2011). Cold exposure reduces serum TG only in HFD‐fed *ApoE* knockout mice without affecting TC or LDL (He et al., 2021), indicating ApoE is required for cold‐induced cholesterol reduction. It also upregulates the activity of lipoprotein lipase (LPL) and ATGL (Weng et al., 2023), two key lipid metabolic enzymes. Angiopoietin‐like protein 4 (ANGPTL4), an adipokine that inhibits LPL activity, plays a critical role in cold‐induced TG reduction. Specifically, cold exposure downregulates ANGPTL4 in BAT, enhancing LPL activity and promoting fatty acid uptake from plasma triglyceride‐rich lipoprotein (TRL) by BAT (Dijk et al., 2015; Singh et al., 2018). In addition, BAT produces the lipokine 12,13‐dihydroxy‐9Z‐octadecenoic acid (12, 13‐dihome), which stimulates membrane translocation of the fatty acid transporters FATP1 and CD36, thereby enhancing FFA uptake and lowering serum TG (Lynes et al., 2017). Cold exposure also increases BAT endothelial cell permeability in mice, facilitating TRL internalization (Bartelt et al., 2011). These mechanisms collectively regulate lipid profiles in obese and hyperlipidemic rodents (Figure 2a), and whether these mechanisms are conserved in humans remains a key focus of future research.

#### 5.1.3 Interaction with anti‐obesity drugs

Cold exposure further modulates the efficacy of anti‐obesity drugs in rodents, with effects dependent on drug type and housing temperature; this interaction has yet to be fully validated in human trials. At conventional housing temperature (22°C), cold acts as a stressor that increases energy expenditure in diet‐induced obese mice relative to thermoneutrality (30°C), confounding assessments of drug‐induced weight loss (Jacobsen et al., 2024). Specifically, 22°C cold exposure enhances the weight‐loss effect of growth differentiation factor 15 (GDF15), diminishes the efficacy of Peptide YY, and exerts no impact on glucagon‐like peptide‐1 (GLP‐1), human fibroblast growth factor 21 (hFGF21), or melanocortin‐4 receptor (MC4R) agonists (Jacobsen et al., 2024). Critically, 22°C cold exposure does not predict drug efficacy in humans who live at thermoneutral conditions, underscoring the need to test anti‐obesity drugs in mice housed near thermoneutrality to avoid overestimating effects, and to validate such interactions in human clinical research.

### 5.2 Human studies

Human studies on cold exposure and obesity focus on translating preclinical findings into clinical relevance, with an emphasis on understanding cold‐induced metabolic responses in diverse human populations, including lean individuals, individuals with overweight, and those with obesity. These studies differ from preclinical rodent models due to the complexity of human physiology, dietary patterns, and environmental adaptations, and they primarily investigate the effects of cold exposure on energy metabolism, body composition, and glucose homeostasis to evaluate its potential as a non‐pharmacological intervention for obesity.

#### 5.2.1 Effects on energy expenditure and body weight

Findings from human studies diverge from rodent models, as there is limited evidence that cold exposure could alter human body weight or composition (McInnis et al., 2020; Yoneshiro et al., 2013), likely due to concurrent increases in energy intake and expenditure that offset each other (McInnis et al., 2025; Unlu et al., 2025). Cold‐induced thermogenesis and elevated energy expenditure (EE) are observed in both lean individuals and those with obesity, but the magnitude of this response is highly dependent on cold exposure parameters, consistent with rodent data showing parameter‐dependent effects.

In lean subjects, acute cold exposure (10°C water‐perfused suit for 1 h, 15°C–16°C water‐perfused blankets for 2 h, or 19°C air with a water‐perfused cooling vest for 2 h) increases EE (Acosta et al., 2018; Mengel et al., 2022; Sellers et al., 2021). However, no EE increase is observed in lean subjects exposed to mild cold (19°C air) without additional local cooling devices (Unlu et al., 2025), highlighting that the use of local cooling devices may contribute to the EE response to mild cold exposure. In addition, cold‐induced EE is influenced by outdoor temperature (Senn et al., 2018) but not by diurnal variations (Acosta et al., 2021), further clarifying the factors that modulate EE responses to cold in lean individuals.

In subjects with obesity, acute cold exposure via a 10°C liquid‐conditioned suit increases EE (McInnis et al., 2025), although some studies report no significant EE changes (Mengel et al., 2022), potentially due to reduced BAT activity in obesity (Brychta et al., 2019; Leitner et al., 2017; Saari et al., 2020; van Marken Lichtenbelt et al., 2009; Yoneshiro & Saito, 2015). Intriguingly, a recent study found comparable BAT activation in BAT‐positive obese and BAT‐positive lean subjects during acute cold exposure (Kulterer et al., 2022), suggesting that discrepancies stem from heterogeneous subject populations in earlier studies. Indeed, in BAT‐positive individuals with overweight or obesity, prolonged mild cold exposure (19°C for 5–8 h) increases resting energy expenditure (REE) by 15% (Chondronikola et al., 2014), and 10 days of intermittent cold exposure (14°C–15°C) enhances BAT activity and EE by approximately 14% (Hanssen et al., 2016).

#### 5.2.2 Effects on glucose metabolism and underlying mechanisms

Parallel to rodent models, acute cold exposure lowers blood glucose levels and enhances insulin sensitivity in individuals with obesity (Hanssen et al., 2016; Mengel et al., 2022). Cold acclimation involving daily shivering (10 days, 1 h/day) further improves glucose tolerance and reduces serum FFAs and TG in subjects with overweight or obesity (Sellers et al., 2024). Notably, a key inter‐model difference emerges here in that cold‐induced improvements in glucose metabolism in humans with obesity stem from enhanced glucose transporter 4 (GLUT4) translocation and increased skeletal muscle glucose uptake, rather than by BAT‐mediated effects (Hanssen et al., 2016; van Beek et al., 2023), highlighting a species‐specific regulatory mechanism.

In conclusion, cold exposure exerts distinct, model‐specific effects on obesity‐related phenotypes. In rodents, sustained cold exposure reliably reduced weight via BAT activation, while also modulating glucose and lipid metabolism and anti‐obesity drug efficacy. In humans, it partially restores thermogenesis, increases EE, and improves glucose metabolism, yet exerts minimal effects on body weight and composition (Table 1). These inter‐model differences emphasize the importance of cautious translation of preclinical findings and highlight the need for targeted human studies to clarify cold exposure's therapeutic potential in obesity management.

## 6 COLD EXPOSURE AND DIABETES

Cold exposure has emerged as a potential non‐pharmacological intervention for diabetes management, with accumulating evidence supporting its beneficial effects on glucose metabolism and insulin sensitivity in both preclinical diabetic models and human patients with type 2 diabetes (T2DM). Below, findings are organized into preclinical (animal) and human studies, with a clear distinction between experimental models and emphasis on underlying mechanisms and inter‐model consistencies or discrepancies.

### 6.1 Preclinical studies

Both acute and long‐term cold exposure exert positive effects on reducing blood glucose levels in streptozotocin‐induced diabetic rodents. For example, cold exposure at 4°C for 24 h or 14 weeks leads to decreased blood glucose levels (Li et al., 2024; Smith & Davidson, 1982), while simultaneously improving glucose tolerance and insulin sensitivity (Li et al., 2024). The cold‐enhanced glucose metabolism observed in these models may be mediated via plasma‐derived extracellular vesicles, which act on multiple tissues including skeletal muscle, liver, WAT, and pancreas through the AKT/IRS1 signaling pathway (Li et al., 2024).

Additionally, prior studies have demonstrated that cold exposure induces GLUT4 translocation in multiple tissues critical for glucose homeostasis, including BAT (Leiria et al., 2019; Nikami et al., 1992), skeletal muscle (Oliveira et al., 2004), and WAT (Gao et al., 2015); this ultimately enhances glucose uptake and lowers blood glucose levels. Notably, increased GLUT4 translocation in skeletal muscle occurs via the AMP‐activated protein kinase (AMPK) signaling pathway (Alvim et al., 2015; Oliveira et al., 2004). Cold exposure also upregulates GLUT4 expression in iWAT, thereby enhancing the anti‐diabetic efficacy of the liver X receptor (LXR) T0901317 agonist in streptozotocin‐induced diabetic mice (Gao et al., 2015), suggesting potential synergistic effects between cold exposure and pharmacological interventions in diabetes management.

### 6.2 Human studies

Human studies on cold exposure and diabetes focus on patients with T2DM, aiming to translate preclinical findings into clinical practice and evaluate the safety and efficacy of cold exposure as a complementary therapeutic strategy. These studies investigate the effects of different cold exposure paradigms (acute vs. chronic, mild vs. moderate) on glucose metabolism and insulin sensitivity, while also exploring factors that may influence treatment outcomes.

In patients with type 2 diabetes (T2DM), cold exposure exhibits promising therapeutic potential, as it improves glucose metabolism and enhances insulin sensitivity (Ivanova & Blondin, 2021) (Table 1). For instance, acute cold exposure, such as a 20‐min cold hip bath, reduces blood glucose levels by ~18% in patients with T2DM (Mooventhan et al., 2020). Similarly, 10 days of cold acclimation at 14°C–15°C improves peripheral insulin sensitivity by 43% in this patient population (Hanssen et al., 2015). However, findings are not universally consistent. A recent study reported that 10 days of mild cold acclimation (16°C–17°C, no obvious shivering) had no effect on insulin sensitivity, postprandial glucose levels, or lipid metabolism in patients with T2DM (Remie et al., 2021). These results suggest that muscle contraction (e.g., shivering) may be a key factor for cold exposure to exert favorable effects on glucose management in human diabetes.

Notably, cold exposure increases GLUT4 translocation in the skeletal muscle of diabetic patients, which may mediate the cold‐induced improvement in insulin sensitivity (Townsend et al., 2023; van Beek et al., 2023), a mechanism consistent with that observed in preclinical rodent models. Additionally, patients with T2DM who exhibit higher BAT activity have better cardiometabolic profiles, such as lower TG, VLDL, and LDL levels, indicating a potential beneficial role of BAT activation in diabetes management and cardiovascular risk reduction (Bonfante et al., 2025).

Collectively, these findings indicate that cold‐induced improvements in glucose metabolism and insulin sensitivity are a complex process, possibly involving multiple tissues (Figure 2b). While preclinical and human studies consistently support the anti‐diabetic potential of cold exposure, discrepancies in human findings highlight the need to optimize cold exposure parameters (e.g., duration, temperature, presence of shivering) to maximize therapeutic efficacy in patients with T2DM.

## 7 COLD EXPOSURE AND CHRONIC INFLAMMATORY DISEASES

Chronic inflammatory diseases, including obesity‐induced metabolic inflammation, metabolic dysfunction‐associated fatty liver disease (MAFLD), cardiovascular diseases, and autoimmune diseases, impose significant health burdens globally. Emerging evidence suggests cold exposure exerts anti‐inflammatory effects by regulating innate and adaptive immune responses, with potential therapeutic implications for these conditions. Below, findings are organized into preclinical studies and human studies, with a focus on underlying mechanisms and clinical relevance.

### 7.1 Preclinical (animal) studies

Preclinical studies utilize diverse rodent and animal models to explore the anti‐inflammatory effects of cold exposure and their underlying molecular mechanisms, covering obesity‐induced inflammation, metabolic dysfunction‐associated fatty liver disease (MAFLD), autoimmune arthritis, and neuroinflammation. These models provide critical insights into tissue‐specific inflammatory regulation and the pathways mediated by cold exposure, laying the foundation for translational research.

#### 7.1.1 Obesity‐induced chronic inflammation and metabolic diseases

Obesity is characterized by chronic low‐grade inflammation, triggered by macrophage infiltration and the local and systemic secretion of pro‐inflammatory cytokines. This inflammation promotes insulin resistance and metabolic disorders such as MAFLD and cardiovascular diseases (Hotamisligil, 2017; Li et al., 2023). Research on MAFLD has investigated various environmental and dietary factors that may modulate its progression, including housing temperature. Nunes, Julia R C et al. examined whether thermoneutral housing (~29°C) accelerates MAFLD in male and female C57Bl/6J mice fed a Western diet high in fat, sugar, and cholesterol (Nunes et al., 2023). Their findings revealed that thermoneutral housing did not accelerate MAFLD in either sex under this obesogenic diet, suggesting that while environmental temperature influences metabolic processes, it may not be a key driver of MAFLD progression under high‐calorie intake. Beyond MAFLD, cold exposure also effectively alleviates obesity‐induced inflammation in diet‐induced obese mice (Sugimoto et al., 2022), as it reduces circulating levels of the pro‐inflammatory cytokine TNF‐α and inhibits the Toll‐like receptor‐mediated inflammatory pathway in the liver (Sugimoto et al., 2022).

Mechanistically, cold activates 12‐lipoxygenase in BAT, releasing the pro‐resolving bioactive lipid MaR2, which increases the proportion of anti‐inflammatory TREM2^+^ macrophages in the liver (Sugimoto et al., 2022). Moreover, cold exposure promotes anti‐inflammatory responses in mesentery adipose tissue, perivascular adipose tissue, and peripheral blood mononuclear cells in ferrets, which may benefit cardiovascular health (Reynés et al., 2017).

#### 7.1.2 Autoimmune disease‐associated inflammation

In addition to attenuating obesity‐induced chronic inflammation, cold exposure has recently been shown to regulate inflammation linked to autoimmune diseases in several rodent models. For example, in antigen‐induced arthritis mice, local cryotherapy (20‐min ice‐cold bag application every 2 h, 2 sessions) alleviates joint inflammation by reducing neutrophil migration and lowering levels of the inflammatory cytokines IL‐1β, IL‐6, and TNF‐α in synovial fluid (Castro et al., 2022). Similarly, in adjuvant‐induced arthritis rats, local cryotherapy (placement in cages lined with ice pops for 30 min, twice daily for 14 days) exerts local and systemic anti‐inflammatory effects, primarily by targeting the IL‐6/IL‐17A pathway (Guillot et al., 2017). It also reduces endothelial activation, immune cell migration, and arterial inflammation, thereby improving vascular health in these rats (Peyronnel et al., 2022). Furthermore, cold exposure attenuates neuroinflammation and delays the onset of experimental autoimmune encephalomyelitis in mice by downregulating Ly6C^high^ monocyte activation and reducing autoreactive T cell priming and cytokine secretion during inflammation (Spiljar et al., 2021).

#### 7.1.3 Mechanisms: Regulation of innate and adaptive immunity

Mechanistically, acute and chronic cold exposure exert distinct effects on immune cells and functions, influencing both innate and adaptive immunity. Acute cold exposure triggers cold stress and may modulate innate and adaptive immunity in healthy young men (Brazaitis et al., 2014). In contrast, chronic cold exposure generally benefits immune function in rodents, with prolonged cold exposure (2°C for over 2 weeks) enhancing murine cellular immune function as evidenced by increased splenic lymphocyte blastogenesis (Xu et al., 1992). Mice continuously exposed to 10°C for 2 weeks show no change in total immune cell numbers but exhibit suppressed innate immune responses characterized by reduced Ly6C^high^ monocytes with lower MHCII expression in bone marrow and blood, which alleviates neuroinflammation (Spiljar et al., 2021). Notably, intermittent cold‐water immersion (12 min at 7°C, four times weekly for 3 weeks) in healthy men has no impact on circulating leukocyte counts (Versteeg et al., 2023), highlighting that cold exposure intensity may be critical for regulating leukocyte numbers.

Cold exposure also exerts regulatory effects on adaptive immunity. In vitro, cold treatment of T cells activates T cell receptor (TCR) signaling and induces widespread intracellular signaling similar to that elicited by soluble antibody stimulation (Ji & Salomon, 2015), suggesting potential utility as a T cell activator. Additionally, both in vitro and in vivo studies show that acute cold stimuli enhance the induction of human FOXP3^+^ regulatory T cells (Tregs) from naïve CD4^+^ T cells (Becker et al., 2019), while chronic cold exposure in mice reduces pro‐inflammatory cytokine production in concanavalin A‐activated splenocytes (Makarova et al., 2005). Collectively, these findings indicate that chronic cold exposure shifts the immune system toward an anti‐inflammatory state.

### 7.2 Human epidemiological studies

Human epidemiological studies complement preclinical findings by exploring associations between cold exposure, inflammation, and chronic diseases, with a focus on cardiovascular health and the role of BAT in modulating cardiometabolic risk. Epidemiological studies demonstrate temperature‐dependent cardiovascular risk, with extreme cold weather associated with higher cardiovascular risk and moderate cold contributing to a considerable attributable risk for cardiovascular diseases (Tian et al., 2016). This may be linked to BAT activation during cold exposure, as a recent study reported that individuals with active BAT have a lower prevalence of cardiometabolic conditions including dyslipidemia, coronary artery disease, cerebrovascular disease, congestive heart failure, and hypertension (Becher et al., 2021).

In summary, cold exposure exhibits promising anti‐inflammatory effects in obesity, arthritis, and neuroinflammation by regulating both innate and adaptive immune responses (Figure 2c). While preclinical studies provide robust mechanistic evidence for its anti‐inflammatory actions, further human research is required to elucidate the underlying molecular pathways in clinical settings and evaluate the feasibility of cold exposure as a complementary intervention for chronic inflammatory diseases.

## 8 COLD EXPOSURE AND CANCER

Obesity is associated with several types of cancer, including breast, gastrointestinal, and thyroid cancer (Lauby‐Secretan et al., 2016), establishing a link between metabolic dysregulation and tumorigenesis. Notably, cold exposure, a key modulator of metabolic and immune function, has been implicated in tumor development and progression, with emerging evidence revealing complex, sometimes contradictory roles. Below, findings are organized into preclinical studies and human studies, focusing on cold exposure's dual effects on tumorigenesis, underlying mechanisms, and potential therapeutic implications.

### 8.1 Preclinical studies

Preclinical studies primarily use tumor‐bearing rodent models to explore the relationship between cold exposure and cancer, dissecting its effects on tumor growth, immune microenvironment, and molecular pathways. These studies have uncovered both pro‐tumorigenic and anti‐tumorigenic effects of cold exposure, highlighting the complexity of this interaction.

#### 8.1.1 Pro‐tumorigenic effects and related mechanisms

Surprisingly, previous epidemiological and preclinical evidence suggests that cold exposure might be linked to enhanced tumor development. For instance, an ecological study demonstrated a correlation between low ambient temperatures and increased cancer incidence (Steiner et al., 2002), providing population‐level support for this association. In tumor‐bearing mice, housing at a sub‐thermoneutral temperature (22°C–23°C) resulted in larger tumor volumes compared to those maintained at a thermoneutral temperature (30°C–31°C), alongside a reduction in CD8^+^ T cells within the tumor microenvironment (Kokolus et al., 2013), indicating impaired immune surveillance, a key factor in tumor progression. Beyond these observations, cold‐sensing receptors, specifically TRPM8 and TRPA1, have also been identified as prognostic biomarkers for cancer (Marini et al., 2023), further linking cold‐sensing pathways to tumor biology. Mechanistically, cold exposure can induce positive regulatory factors involved in tumorigenesis, such as the cold‐inducible RNA‐binding protein (CIRP) and RNA‐binding motif protein 3 (RBM3) (Zhu et al., 2016). These factors have been implicated in promoting cell proliferation, survival, and metastasis in various cancer types, providing a potential molecular link between cold exposure and tumor progression.

#### 8.1.2 Anti‐tumorigenic effects and related mechanisms

In contrast to its pro‐tumorigenic potential, recent preclinical studies have demonstrated that cold exposure can inhibit tumor growth in mice by depriving tumor cells of glucose, a critical nutrient for their rapid proliferation (Figure 2d). For example, 20 days of cold exposure suppressed tumor growth and improved survival in tumor‐bearing mice implanted with various murine and human tumor cells, including breast cancer, colon cancer, pancreatic ductal adenocarcinoma, fibrosarcoma, and melanoma cells (Seki et al., 2022).

The anti‐tumor mechanism is primarily mediated by BAT activation, as cold exposure induces UCP1‐dependent BAT thermogenesis, which increases systemic glucose consumption, thereby reducing glucose uptake in tumors and lowering circulating blood glucose levels (Seki et al., 2022). Additionally, cold exposure inhibits the glycolytic pathway, a key energy‐generating process for tumor cells, inducing metabolic reprogramming within tumors that impairs their growth and survival (Seki et al., 2022). This mechanism was further validated by experiments showing that surgical removal of BAT combined with a high‐glucose diet reversed the cold‐induced tumor growth inhibition, emphasizing the essential roles of BAT and glucose limitation in the cold‐mediated anti‐cancer effect (Seki et al., 2022).

#### 8.1.3 Interaction with tumor vaccines

Beyond direct metabolic effects, cold exposure can enhance the efficacy of tumor vaccines, providing additional support for its potential in cancer therapy. Specifically, cold exposure promoted the proliferation of CD8^+^ T cells and memory T cells, thereby enhancing the protective efficacy of tumor vaccines against 4 T1 breast cancer cells or CT26 colon cancer cells (Ye et al., 2025). These findings suggest that cold exposure could be used as an adjuvant strategy to improve the effectiveness of cancer immunotherapy (Seki et al., 2022).

### 8.2 Human studies

Human research on cold exposure and cancer remains limited, with only a pilot study providing preliminary evidence for its anti‐tumor potential. In cancer patients with Hodgkin's lymphoma, mild cold exposure reduced glucose uptake in tumor tissue (Seki et al., 2022), mirroring the glucose‐deprivation mechanism observed in preclinical models. However, no additional human studies have been conducted to validate these findings, and the pro‐tumorigenic correlates observed in epidemiological studies require further investigation to clarify their clinical relevance.

## 9 POTENTIAL RISKS ASSOCIATED WITH COLD EXPOSURE

While cold exposure holds therapeutic potential for metabolic, inflammatory, and even cancer‐related conditions, it is also associated with several potential health risks, including stress responses, cardiovascular effects, transient cognitive impairment, and disrupted reproductive endocrine function. Importantly, two distinct cold exposure paradigms, whole body cooling and cold pressor tests, elicit unique physiological responses, and their effects must not be conflated when evaluating cold‐related risks.

Whole‐body cooling, the primary paradigm relevant to cold exposure as a therapeutic intervention, can induce transient physiological responses in healthy humans. For example, acute whole‐body cold exposure via 14°C cold‐water immersion (either 10 min per session or 20 min intermittently for 6 sessions) triggers a marked release of stress hormones (e.g. cortisol, epinephrine, and norepinephrine) immediately after exposure and even 2 days post‐exposure (Eimonte et al., 2022). Similarly, preclinical studies in rats show that acute mild whole‐body cold exposure (15 °C and 18°C for 24 h) (Chen, Wu, et al., 2021) or 10 °C for 4 days (Yoshimoto et al., 2025) elicits elevated blood pressure and increased renal sympathetic nerve activity. These response are adaptive, serving to maintain core body temperature, and do not equate to inherent health risks.

In contrast, the cold pressor test represents a localized acute stressor that induces distinct physiological effects not representative of whole‐body cooling. For example, in patients with diabetes or glucose intolerance who exhibit platelet activation, 15‐min forearm immersion in melting ice increased thrombotic activity (Maiello et al., 1988), potentially raising cardiovascular disease risk. This finding is specific to the cold pressor test and cannot be extrapolated to whole‐body cooling paradigms.

Beyond cardiovascular‐related responses, cold exposure has been associated with transient changes in cognitive function and reproductive endocrine parameters in specific contexts. For instance, females exposed to 10°C cold air for 140 min demonstrated impaired reaction time during cognitive function testing, as measured by wearable electrodermal activity (EDA) and electrocardiography (ECG) devices (Kong et al., 2025). This impairment is temporary, likely linked to the body prioritizing thermoregulation over cognitive processes, rather than a long‐term health risk. In preclinical studies, chronic intermittent cold exposure (10°C, 4 h/day for 2 weeks) in Sprague–Dawley (SD) female rats induced peri‐ovarian adipose tissue browning, but also caused local microvascular circulatory disturbances in ovarian and uterine tissue (Wang et al., 2020), abnormal follicular development, and impaired ovarian function (Zhang et al., 2021) These findings are preliminary and require translational research to determine their relevance to humans.

Collectively, these findings underscore the importance of distinguishing between cold exposure paradigms. While specific contexts may elicit transient physiological responses, these are not universal risks. Future human research should prioritize clarifying paradigm‐specific effects, particularly in vulnerable populations such as individuals with metabolic disorders and women.

## 10 CONCLUSIONS AND PERSPECTIVES

**FIGURE 3:**
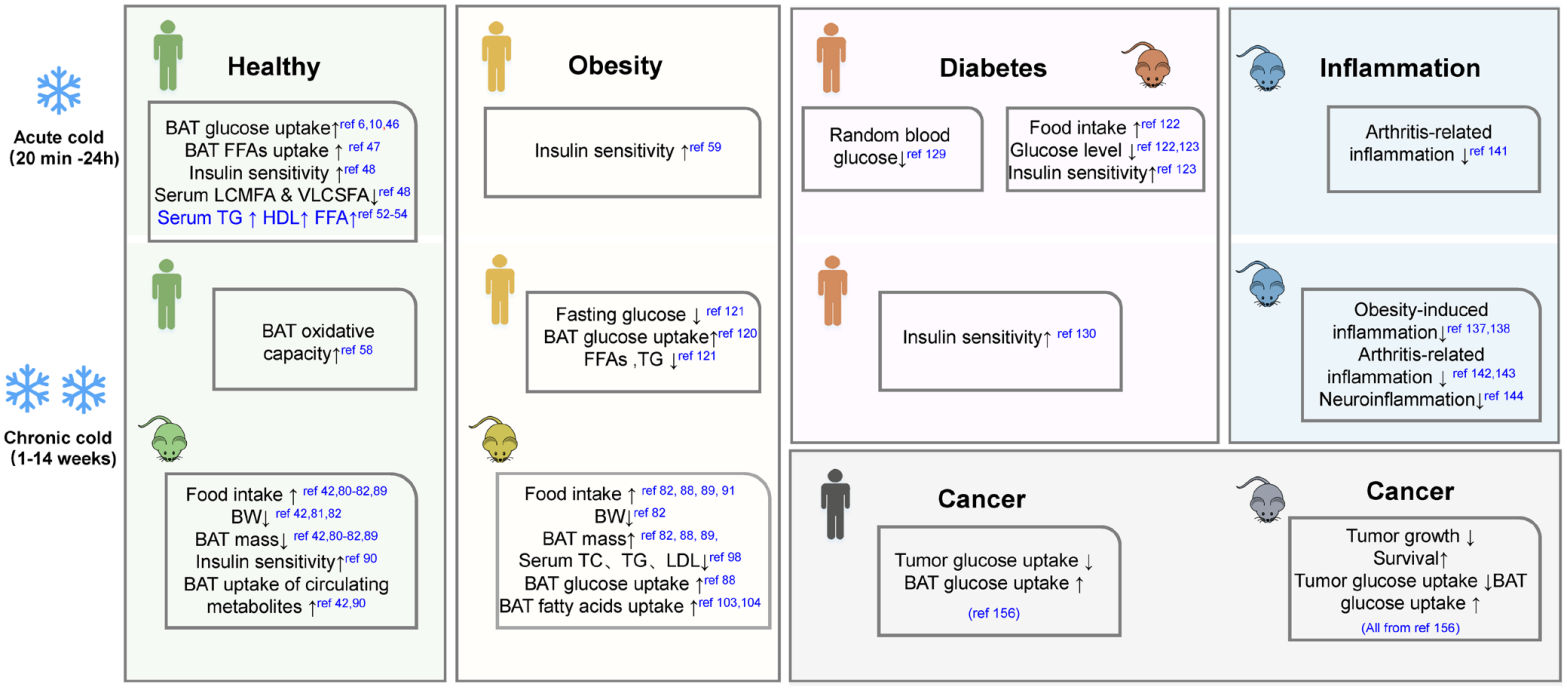
Summary of the beneficial effects of cold exposure on health, obesity, diabetes, inflammation, and cancer. Cold exposure increases brown adipose tissue (BAT) activity and enhances glucose and lipid metabolism in both healthy humans and rodents. In obese and diabetic conditions, cold exposure can also improve glucose and lipid metabolism and insulin sensitivity. Moreover, in murine models, cold exposure has been demonstrated to modulate inflammation, including obesity‐induced inflammation, arthritis‐related inflammation, and neuroinflammation; yet, its translational potential to humans remains uncertain. Additionally, recent research suggests that cold exposure may inhibit tumor growth, possibly through mechanisms involving increased glucose uptake by BAT and decreased glucose availability for tumors. BAT, brown adipose tissue; EE, energy expenditure; REE, resting energy expenditure.

The rising prevalence of obesity and related comorbidities has underscored the urgency of developing novel therapeutic strategies. The discovery of BAT in adult humans and cold‐induced browning of WAT have sparked extensive research on the therapeutic potential of cold exposure for treating obesity and metabolic disorders. Emerging evidence demonstrates that cold exposure reduces serum lipid levels and enhances insulin sensitivity in both healthy individuals and patients with obesity or T2DM (Figure 3). Preclinical studies further reveal its role in mitigating neuroinflammation and inhibiting tumor growth, suggesting broad applications in managing metabolic diseases, inflammatory conditions, and even cancer (Figure 3).

Despite these promising attributes, several critical factors must be addressed for the clinical translation of cold exposure‐based therapies. First, the absence of standardized guidelines for the optimal intensity, duration, and frequency of cold exposure across patient populations warrants further investigation. For example, individuals with obesity exhibit variability in body mass index (BMI) and fat distribution, which can influence their response to cold (Tashani et al., 2017). Moreover, patients with diabetes may experience altered cold perception due to diabetic peripheral neuropathy (Fang et al., 2020), complicating the standardization of treatment protocols. In addition, genetic variation in cold receptors differs substantially among populations (Schutz et al., 2014). While the identification of cold receptors, primarily from preclinical studies, has clarified how cold exposure acts on sensory neurons, the in vivo molecular mechanisms underlying its metabolic and anti‐inflammatory effects remain incompletely understood. Further research is needed to unravel the cellular and molecular pathways through which cold exposure modulates adipose tissue function, muscle metabolism, and endocrine signaling.

Second, limitations of current cooling devices present practical barriers to clinical application. Three distinct cooling techniques are currently used to activate human BAT (Chen et al., 2016): (1) maintaining a fixed ambient room temperature, a mild cooling strategy that minimizes muscle shivering while activating BAT; (2) using a water‐cooling blanket or personalized suit set to 1–2°C above the symptomatic shivering threshold; and (3) immersing the participant's arms or legs in cold water or ice. The water‐cooling blanket or personalized suit method likely maximizes BAT activation (Chen et al., 2016), but it also enhances thermogenesis in muscles and other tissues. A major drawback of these devices, however, is their potential to cause uneven cooling and frequently trigger shivering (Chen, Malhotra, et al., 2021). Cold water or ice immersing, by contrast, can induce pain‐mediated sympathetic activation, rendering it intolerable for prolonged use.

Third, a more comprehensive understanding of human BAT activation and thermogenesis mechanisms is essential. Historically, ^18^F‐fluorodeoxyglucose positron emission tomography/computed tomography (^18^F‐FDG PET/CT) has been the most widely used imaging technique for evaluating BAT mass and activation (Ong et al., 2018). While this method has yielded valuable insights, its ability to accurately reflect BAT thermogenesis is debated because preclinical studies have shown that BAT utilizes not only glucose but also FFAs and BCAAs for energy production (Carpentier & Blondin, 2023). To address this limitation, isotope‐tracing studies are needed to precisely define the role of glucose in human BAT thermogenesis. Magnetic resonance imaging (MRI) represents a highly promising alternative due to its non‐invasive nature and lack of radiation exposure. Several MRI studies have successfully quantified dynamic changes in human BAT volume, activation status, and fat content following cold exposure (Chen et al., 2013; Stahl et al., 2017). More recently, a non‐invasive, radiation‐free metabolic MRI technique based on creatine chemical exchange saturation transfer (Cr‐CEST) contrast has been validated for evaluating in vivo BAT activity in both rodents and humans, offering a novel approach for BAT functional mapping (Cai et al., 2024).

Finally, several key research avenues need to be explored to fully realize the potential of cold exposure as a therapeutic strategy. Long‐term follow‐up studies are required to assess the sustainability of cold‐induced metabolic improvements and determine whether initial benefits persist over time. Combination therapies pairing cold exposure with established interventions such as exercise programs or anti‐obesity and anti‐diabetic medications may also represent a viable strategy for metabolic diseases. In addition, investigating regulators of BAT activation and cold‐responsive signaling molecules in preclinical models provides promising directions for identifying novel therapeutic targets. Furthermore, future research could integrate cold exposure with lifestyle modifications such as fasting and exercise to maximize its metabolic benefits (Asghari Alashti et al., 2025).

In summary, cold exposure holds substantial promise as a therapeutic intervention for obesity, metabolic disorders, inflammation, and cancer. However, addressing gaps in standardized protocols, advancing cooling device technology, refining BAT imaging methods, and unraveling the underlying molecular mechanisms will be critical to translating its preclinical potential into safe, effective clinical treatments.

## AUTHOR CONTRIBUTIONS

**Jiale Dang:** Data curation; visualization. **Dan Gao:** Conceptualization. **Helen R. Griffiths:** Conceptualization. **Rui Guo:** Data curation. **Xirong Li:** Data curation; visualization. **Shemin Lu:** Conceptualization.

## FUNDING INFORMATION

This work is supported the National Natural Science Foundation of China (NSFC) (Grant No. 81873665 to Dan Gao, 82102726 to Rui Guo), Shaanxi Province Science and Technology Research and Development Program (Grant No. 2024JC‐YBMS‐608 to Dan Gao).

## CONFLICT OF INTEREST STATEMENT

The authors declare no conflicts of interest.

## Supporting information


Table S1.

## Data Availability

The authors have nothing to report.

## References

[phy270838-bib-0001] Acosta, F. M. , Martinez‐Tellez, B. , Sanchez‐Delgado, G. , A. Alcantara, J. M. , Acosta‐Manzano, P. , Morales‐Artacho, A. J. , & R. Ruiz, J. (2018). Physiological responses to acute cold exposure in young lean men. PLoS One, 13(5), e0196543. 29734360 10.1371/journal.pone.0196543 PMC5937792

[phy270838-bib-0002] Acosta, F. M. , Sanchez‐Delgado, G. , Martinez‐Tellez, B. , Alcantara, J. M. A. , Llamas‐Elvira, J. M. , & Ruiz, J. R. (2021). Diurnal variations of cold‐induced thermogenesis in young, healthy adults: A randomized crossover trial. Clinical Nutrition, 40(10), 5311–5321. 34536639 10.1016/j.clnu.2021.08.010

[phy270838-bib-0003] Aldiss, P. , Lewis, J. E. , Lupini, I. , Bloor, I. , Chavoshinejad, R. , Boocock, D. J. , Miles, A. K. , Ebling, F. J. P. , Budge, H. , & Symonds, M. E. (2022). Cold exposure drives weight gain and adiposity following chronic suppression of Brown adipose tissue. International Journal of Molecular Sciences, 23(3), 1869. 35163791 10.3390/ijms23031869 PMC8836787

[phy270838-bib-0004] Allan, R. , Malone, J. , Alexander, J. , Vorajee, S. , Ihsan, M. , Gregson, W. , Kwiecien, S. , & Mawhinney, C. (2022). Cold for centuries: A brief history of cryotherapies to improve health, injury and post‐exercise recovery. European Journal of Applied Physiology, 122(5), 1153–1162. 35195747 10.1007/s00421-022-04915-5 PMC9012715

[phy270838-bib-0005] Alvim, R. O. , Cheuhen, M. A. R. C. E. L. R. , Machado, S. I. L. M. A. R. A. R. , Sousa, A. N. D. R. É. G. U. S. T. A. V. O. P. , & Santos, P. A. U. L. O. C. J. L. (2015). General aspects of muscle glucose uptake. Anais da Academia Brasileira de Ciências, 87(1), 351–368. 25761221 10.1590/0001-3765201520140225

[phy270838-bib-0006] Anunciado‐Koza, R. , Ukropec, J. , Koza, R. A. , & Kozak, L. P. (2008). Inactivation of UCP1 and the glycerol phosphate cycle synergistically increases energy expenditure to resist diet‐induced obesity. The Journal of Biological Chemistry, 283(41), 27688–27697. 18678870 10.1074/jbc.M804268200 PMC2562063

[phy270838-bib-0007] Asghari Alashti, F. , Goliaei, B. , Asghari Alashti, F. , Goliaei, B. , Asghari Alashti, F. , & Goliaei, B. (2025). Rethinking fat Browning: Uncovering new molecular insights into the synergistic roles of fasting, exercise, and cold exposure. European Journal of Pharmacology, 998, 177651. 40274179 10.1016/j.ejphar.2025.177651

[phy270838-bib-0008] Bardova, K. , Janovska, P. , Vavrova, A. , Kopecky, J. , & Zouhar, P. (2024). Adaptive induction of nonshivering thermogenesis in muscle rather than Brown fat could counteract obesity. Physiological Research, 73(S1), S279–S294. 38752772 10.33549/physiolres.935361 PMC11412341

[phy270838-bib-0009] Bartelt, A. , Bruns, O. T. , Reimer, R. , Hohenberg, H. , Ittrich, H. , Peldschus, K. , Kaul, M. G. , Tromsdorf, U. I. , Weller, H. , Waurisch, C. , Eychmüller, A. , Gordts, P. L. S. M. , Rinninger, F. , Bruegelmann, K. , Freund, B. , Nielsen, P. , Merkel, M. , & Heeren, J. (2011). Brown adipose tissue activity controls triglyceride clearance. Nature Medicine, 17(2), 200–205. 10.1038/nm.2297 21258337

[phy270838-bib-0010] Bautista, D. M. , Siemens, J. , Glazer, J. M. , Tsuruda, P. R. , Basbaum, A. I. , Stucky, C. L. , Jordt, S. E. , & Julius, D. (2007). The menthol receptor TRPM8 is the principal detector of environmental cold. Nature, 448(7150), 204–208. 17538622 10.1038/nature05910

[phy270838-bib-0011] Becher, T. , Palanisamy, S. , Kramer, D. J. , Eljalby, M. , Marx, S. J. , Wibmer, A. G. , Butler, S. D. , Jiang, C. S. , Vaughan, R. , Schöder, H. , Mark, A. , & Cohen, P. (2021). Brown adipose tissue is associated with cardiometabolic health. Nature Medicine, 27(1), 58–65. 10.1038/s41591-020-1126-7 PMC8461455 33398160

[phy270838-bib-0012] Becker, M. , Serr, I. , Salb, V. K. , Ott, V. B. , Mengel, L. , Blüher, M. , Weigmann, B. , Hauner, H. , Tschöp, M. H. , & Daniel, C. (2019). Short‐term cold exposure supports human Treg induction in vivo. Molecular Metabolism, 28, 73–82. 31427184 10.1016/j.molmet.2019.08.002 PMC6822223

[phy270838-bib-0013] Berry, D. C. , Jiang, Y. , & Graff, J. M. (2016). Mouse strains to study cold‐inducible beige progenitors and beige adipocyte formation and function. Nature Communications, 7, 10184. 10.1038/ncomms10184 PMC4728429 26729601

[phy270838-bib-0014] Betz, M. J. , & Enerback, S. (2018). Targeting thermogenesis in brown fat and muscle to treat obesity and metabolic disease. Nature Reviews. Endocrinology, 14(2), 77–87. 10.1038/nrendo.2017.132 29052591

[phy270838-bib-0015] Blondin, D. P. , Labbé, S. M. , Phoenix, S. , Guérin, B. , Turcotte, É. E. , Richard, D. , Carpentier, A. C. , & Haman, F. (2015). Contributions of white and brown adipose tissues and skeletal muscles to acute cold‐induced metabolic responses in healthy men. The Journal of Physiology, 593(3), 701–714. 25384777 10.1113/jphysiol.2014.283598 PMC4324714

[phy270838-bib-0016] Blondin, D. P. , Nielsen, S. , Kuipers, E. N. , Severinsen, M. C. , Jensen, V. H. , Miard, S. , Jespersen, N. Z. , Kooijman, S. , Boon, M. R. , Fortin, M. , Phoenix, S. , Frisch, F. , Guérin, B. , & Turcotte, É. E. (2020). Human Brown adipocyte thermogenesis is driven by beta2‐AR stimulation. Cell Metabolism, 32(2), 287–300. 32755608 10.1016/j.cmet.2020.07.005

[phy270838-bib-0017] Blondin, D. P. , Tingelstad, H. C. , Noll, C. , Frisch, F. , Phoenix, S. , Guérin, B. , Turcotte, É. E. , Richard, D. , Haman, F. , & Carpentier, A. C. (2017). Dietary fatty acid metabolism of brown adipose tissue in cold‐acclimated men. Nature Communications, 8, 14146. 10.1038/ncomms14146 PMC5290270 28134339

[phy270838-bib-0018] Bobbioni‐Harsch, E. , Assimacopoulos‐Jeannet, F. , & Jeanrenaud, B. (1994). Modifications of glucose and lipid metabolism in cold‐acclimated lean and genetically obese rats. Journal of Applied Physiology (1985), 76(3), 1106–1112. 10.1152/jappl.1994.76.3.1106 8005851

[phy270838-bib-0019] Bonfante, I. L. P. , Segantim, H. D. S. , Mendonça, K. N. S. , de Oliveira, M. A. B. , Monfort‐Pires, M. , Duft, R. G. , da Silva Mateus, K. C. , Chacon‐Mikahil, M. P. T. , Ramos, C. D. , Velloso, L. A. , & Cavaglieri, C. R. (2025). Better cardiometabolic/inflammatory profile is associated with differences in the supraclavicular adipose tissue activity of individuals with T2DM. Endocrine, 87(3), 1011–1021. 39627400 10.1007/s12020-024-04122-6

[phy270838-bib-0020] Bornstein, M. R. , Neinast, M. D. , Zeng, X. , Chu, Q. , Axsom, J. , Thorsheim, C. , Li, K. , Blair, M. C. , Rabinowitz, J. D. , & Arany, Z. (2023). Comprehensive quantification of metabolic flux during acute cold stress in mice. Cell Metabolism, 35(11), 2077–2092. 37802078 10.1016/j.cmet.2023.09.002 PMC10840821

[phy270838-bib-0021] Brauchi, S. , Orta, G. , Salazar, M. , Rosenmann, E. , & Latorre, R. (2006). A hot‐sensing cold receptor: C‐terminal domain determines thermosensation in transient receptor potential channels. The Journal of Neuroscience, 26(18), 4835–4840. 16672657 10.1523/JNEUROSCI.5080-05.2006 PMC6674176

[phy270838-bib-0022] Brazaitis, M. , Eimantas, N. , & Daniuseviciute, L. (2014). Two strategies for response to 14 degrees C cold‐water immersion: Is there a difference in the response of motor, cognitive, immune and stress markers? PLoS One, 9(9), e109020. 25275647 10.1371/journal.pone.0109020 PMC4183517

[phy270838-bib-0023] Brychta, R. J. , Huang, S. , Wang, J. , Leitner, B. P. , Hattenbach, J. D. , Bell, S. L. , Fletcher, L. A. , Perron Wood, R. , Idelson, C. R. , Duckworth, C. J. , McGehee, S. , Courville, A. B. , Bernstein, S. B. , Reitman, M. L. , Cypess, A. M. , & Chen, K. Y. (2019). Quantification of the capacity for cold‐induced thermogenesis in Young men with and without obesity. The Journal of Clinical Endocrinology and Metabolism, 104(10), 4865–4878. 31150063 10.1210/jc.2019-00728 PMC6733495

[phy270838-bib-0024] Cai, Z. , Zhong, Q. , Feng, Y. , Wang, Q. , Zhang, Z. , Wei, C. , Yin, Z. , Liang, C. , Liew, C. W. , Kazak, L. , Cypess, A. M. , Liu, Z. , & Cai, K. (2024). Non‐invasive mapping of brown adipose tissue activity with magnetic resonance imaging. Nature Metabolism, 6(7), 1367–1379. 10.1038/s42255-024-01082-z PMC11272596 39054361

[phy270838-bib-0025] Camacho, S. , Michlig, S. , de Senarclens‐Bezençon, C. , Meylan, J. , Meystre, J. , Pezzoli, M. , Markram, H. , & le Coutre, J. (2015). Anti‐obesity and anti‐hyperglycemic effects of cinnamaldehyde via altered ghrelin secretion and functional impact on food intake and gastric emptying. Scientific Reports, 5, 7919. 25605129 10.1038/srep07919 PMC4300502

[phy270838-bib-0026] Cannon, B. , de Jong, J. M. A. , Fischer, A. W. , Nedergaard, J. , & Petrovic, N. (2020). Human brown adipose tissue: Classical brown rather than brite/beige? Experimental Physiology, 105(8), 1191–1200. 32378255 10.1113/EP087875

[phy270838-bib-0027] Carpentier, A. C. , & Blondin, D. P. (2023). Human brown adipose tissue is not enough to combat cardiometabolic diseases. The Journal of Clinical Investigation, 133(23), e175288. 38038130 10.1172/JCI175288 PMC10688973

[phy270838-bib-0028] Castellani, J. W. , & Young, A. J. (2016). Human physiological responses to cold exposure: Acute responses and acclimatization to prolonged exposure. Autonomic Neuroscience, 196, 63–74. 26924539 10.1016/j.autneu.2016.02.009

[phy270838-bib-0029] Castro, P. , Castro, P. A. T. S. , Barbosa, G. M. , Machanocker, D. H. , Peres, R. S. , Cunha, T. M. , Cunha, J. E. , Oliveira, F. F. B. , Ramalho, F. S. , Russo, T. L. , Cunha, F. Q. , & Salvini, T. F. (2022). Clinical‐like cryotherapy in acute knee arthritis of the knee improves inflammation signs, pain, joint swelling, and motor performance in mice. PLoS One, 17(1), e0261667. 35061737 10.1371/journal.pone.0261667 PMC8782531

[phy270838-bib-0030] Chen, C. W. , Wu, C. H. , Liou, Y. S. , Kuo, K. L. , Chung, C. H. , Lin, Y. T. , Kuo, T. B. J. , & Yang, C. C. H. (2021). Roles of cardiovascular autonomic regulation and sleep patterns in high blood pressure induced by mild cold exposure in rats. Hypertension Research, 44(6), 662–673. 33742169 10.1038/s41440-021-00619-z

[phy270838-bib-0031] Chen, K. Y. , Cypess, A. M. , Laughlin, M. R. , Haft, C. R. , Hu, H. H. , Bredella, M. A. , Enerbäck, S. , Kinahan, P. E. , Lichtenbelt, W. M. , Lin, F. I. , Sunderland, J. J. , Virtanen, K. A. , & Wahl, R. L. (2016). Brown adipose reporting criteria in imaging STudies (BARCIST 1.0): Recommendations for standardized FDG‐PET/CT experiments in humans. Cell Metabolism, 24(2), 210–222. 27508870 10.1016/j.cmet.2016.07.014 PMC4981083

[phy270838-bib-0032] Chen, T. , Malhotra, P. , Khameraj, A. , Ong‐Bello, N. , Vyas, P. P. , Rasul, R. , Schwartz, R. M. , & Farber, B. F. (2021). Cooling blankets in hospitalized patients: Time to reevaluate. The American Journal of the Medical Sciences, 362(6), 601–605. 34161829 10.1016/j.amjms.2021.06.009

[phy270838-bib-0033] Chen, Y. C. , Iris Chen, Y.‐C. , Cypess, A. M. , Palmer, M. , Kolodny, G. , Kahn, C. R. , & Kwong, K. K. (2013). Measurement of human brown adipose tissue volume and activity using anatomic MR imaging and functional MR imaging. Journal of Nuclear Medicine, 54(9), 1584–1587. 23868958 10.2967/jnumed.112.117275 PMC4167352

[phy270838-bib-0034] Chondronikola, M. , Volpi, E. , Børsheim, E. , Porter, C. , Annamalai, P. , Enerbäck, S. , Lidell, M. E. , Saraf, M. K. , Labbe, S. M. , Hurren, N. M. , Yfanti, C. , Chao, T. , Andersen, C. R. , Cesani, F. , Hawkins, H. , & Sidossis, L. S. (2014). Brown adipose tissue improves whole‐body glucose homeostasis and insulin sensitivity in humans. Diabetes, 63(12), 4089–4099. 25056438 10.2337/db14-0746 PMC4238005

[phy270838-bib-0035] Clemmensen, C. , Jall, S. , Kleinert, M. , Quarta, C. , Gruber, T. , Reber, J. , Sachs, S. , Fischer, K. , Feuchtinger, A. , Karlas, A. , Simonds, S. E. , Grandl, G. , Loher, D. , Sanchez‐Quant, E. , Keipert, S. , Jastroch, M. , Hofmann, S. M. , Nascimento, E. B. M. , Schrauwen, P. , … Tschöp, M. H. (2018). Coordinated targeting of cold and nicotinic receptors synergistically improves obesity and type 2 diabetes. Nature Communications, 9(1), 4304. 10.1038/s41467-018-06769-y PMC6199300 30353008

[phy270838-bib-0036] Cohen, P. , & Kajimura, S. (2021). The cellular and functional complexity of thermogenic fat. Nature Reviews. Molecular Cell Biology, 22(6), 393–409. 33758402 10.1038/s41580-021-00350-0 PMC8159882

[phy270838-bib-0037] Cousin, B. , Cinti, S. , Morroni, M. , Raimbault, S. , Ricquier, D. , Pénicaud, L. , & Casteilla, L. (1992). Occurrence of brown adipocytes in rat white adipose tissue: Molecular and morphological characterization. Journal of Cell Science, 103(4), 931–942. 1362571 10.1242/jcs.103.4.931

[phy270838-bib-0038] Cypess, A. M. , Lehman, S. , Williams, G. , Tal, I. , Rodman, D. , Goldfine, A. B. , Kuo, F. C. , Palmer, E. L. , Tseng, Y. H. , Doria, A. , Kolodny, G. M. , & Kahn, C. R. (2009). Identification and importance of brown adipose tissue in adult humans. The New England Journal of Medicine, 360(15), 1509–1517. 19357406 10.1056/NEJMoa0810780 PMC2859951

[phy270838-bib-0039] da Silva, J. T. , Cella, P. S. , Testa, M. T. J. , Perandini, L. A. , Festuccia, W. T. , Deminice, R. , & Chimin, P. (2020). Mild‐cold water swimming does not exacerbate white adipose tissue browning and brown adipose tissue activation in mice. Journal of Physiology and Biochemistry, 76(4), 663–672. 33051822 10.1007/s13105-020-00771-z

[phy270838-bib-0040] de Jong, J. M. A. , Cannon, B. , Nedergaard, J. , Wolfrum, C. , & Petrovic, N. (2020). Reply to ‘confounding issues in the ‘humanized’ brown fat of mice’. Nature Metabolism, 2(4), 305–306. 10.1038/s42255-020-0193-x 32694605

[phy270838-bib-0041] de Jong, J. M. A. , Sun, W. , Pires, N. D. , Frontini, A. , Balaz, M. , Jespersen, N. Z. , Feizi, A. , Petrovic, K. , Fischer, A. W. , Bokhari, M. H. , Niemi, T. , Nuutila, P. , Cinti, S. , Nielsen, S. , Scheele, C. , Virtanen, K. , Cannon, B. , Nedergaard, J. , Wolfrum, C. , & Petrovic, N. (2019). Human brown adipose tissue is phenocopied by classical brown adipose tissue in physiologically humanized mice. Nature Metabolism, 1(8), 830–843. 10.1038/s42255-019-0101-4 32694768

[phy270838-bib-0042] Dhaka, A. , Murray, A. N. , Mathur, J. , Earley, T. J. , Petrus, M. J. , & Patapoutian, A. (2007). TRPM8 is required for cold sensation in mice. Neuron, 54(3), 371–378. 17481391 10.1016/j.neuron.2007.02.024

[phy270838-bib-0043] Dijk, W. , Heine, M. , Vergnes, L. , Boon, M. R. , Schaart, G. , Hesselink, M. K. C. , Reue, K. , van Marken Lichtenbelt, W. D. , Olivecrona, G. , Rensen, P. C. N. , Heeren, J. , & Kersten, S. (2015). ANGPTL4 mediates shuttling of lipid fuel to brown adipose tissue during sustained cold exposure. eLife, 4, 4. 10.7554/eLife.08428 PMC4709329 26476336

[phy270838-bib-0044] Eimonte, M. , Eimantas, N. , Baranauskiene, N. , Solianik, R. , & Brazaitis, M. (2022). Kinetics of lipid indicators in response to short‐ and long‐duration whole‐body, cold‐water immersion. Cryobiology, 109, 62–71. 36150503 10.1016/j.cryobiol.2022.09.003

[phy270838-bib-0045] Fang, W. C. , Chou, K. M. , Sun, C. Y. , Lee, C. C. , Wu, I. W. , Chen, Y. C. , & Pan, H. C. (2020). Thermal perception abnormalities can predict diabetic kidney disease in type 2 diabetes mellitus patients. Kidney & Blood Pressure Research, 45(6), 926–938. 33053551 10.1159/000510479

[phy270838-bib-0046] Finlin, B. S. , Memetimin, H. , Confides, A. L. , Kasza, I. , Zhu, B. , Vekaria, H. J. , Harfmann, B. , Jones, K. A. , Johnson, Z. R. , Westgate, P. M. , Alexander, C. M. , Sullivan, P. G. , Dupont‐Versteegden, E. E. , & Kern, P. A. (2018). Human adipose beiging in response to cold and mirabegron. JCI Insight, 3(15), e121510. 30089732 10.1172/jci.insight.121510 PMC6129119

[phy270838-bib-0047] Gao, M. , Zhang, C. , Ma, Y. , & Liu, D. (2015). Cold exposure improves the anti‐diabetic effect of T0901317 in Streptozotocin‐induced diabetic mice. The AAPS Journal, 17(3), 700–710. 25739819 10.1208/s12248-015-9746-4 PMC4406970

[phy270838-bib-0048] Garcia‐Oreja, S. , Álvaro‐Afonso, F. J. , Tardáguila‐García, A. , López‐Moral, M. , García‐Madrid, M. , & Lázaro‐Martínez, J. L. (2022). Efficacy of cryotherapy for plantar warts: A systematic review and meta‐analysis. Dermatologic Therapy, 35(6), e15480. 35365922 10.1111/dth.15480 PMC9285476

[phy270838-bib-0049] Geerling, J. J. , Boon, M. R. , Kooijman, S. , Parlevliet, E. T. , Havekes, L. M. , Romijn, J. A. , Meurs, I. M. , & Rensen, P. C. N. (2014). Sympathetic nervous system control of triglyceride metabolism: Novel concepts derived from recent studies. Journal of Lipid Research, 55(2), 180–189. 24285857 10.1194/jlr.R045013 PMC3886657

[phy270838-bib-0050] Guillot, X. , Martin, H. , Seguin‐Py, S. , Maguin‐Gaté, K. , Moretto, J. , Totoson, P. , Wendling, D. , Demougeot, C. , & Tordi, N. (2017). Local cryotherapy improves adjuvant‐induced arthritis through down‐regulation of IL‐6 / IL‐17 pathway but independently of TNFα. PLoS One, 12(7), e0178668. 28759646 10.1371/journal.pone.0178668 PMC5536266

[phy270838-bib-0051] Gupta, R. K. (2023). Human brown fat and metabolic disease: A heated debate. The Journal of Clinical Investigation, 133(23), e176678. 38038128 10.1172/JCI176678 PMC10688977

[phy270838-bib-0052] Haman, F. , Mantha, O. L. , Cheung, S. S. , DuCharme, M. B. , Taber, M. , Blondin, D. P. , & McGarr, G. W. (2016). Oxidative fuel selection and shivering thermogenesis during a 12‐ and 24‐h cold‐survival simulation. Journal of Applied Physiology (1985), 120(6), 640–648. 10.1152/japplphysiol.00540.2015 26718783

[phy270838-bib-0053] Hanssen, M. J. , Hoeks, J. , Brans, B. , & Van Der Lans, A. (2015). Short‐term cold acclimation improves insulin sensitivity in patients with type 2 diabetes mellitus. Nature Medicine, 21(8), 863–865. 10.1038/nm.3891 26147760

[phy270838-bib-0054] Hanssen, M. J. , van der Lans, A. A. , Brans, B. , Hoeks, J. , Jardon, K. M. , & Schaart, G. (2016). Short‐term cold acclimation recruits Brown adipose tissue in obese humans. Diabetes, 65(5), 1179–1189. 26718499 10.2337/db15-1372

[phy270838-bib-0055] Harri, M. , Dannenberg, T. , Oksanen‐Rossi, R. , Hohtola, E. , & Sundin, U. (1984). Related and unrelated changes in response to exercise and cold in rats: A reevaluation. Journal of Applied Physiology: Respiratory, Environmental and Exercise Physiology, 57(5), 1489–1497. 6520042 10.1152/jappl.1984.57.5.1489

[phy270838-bib-0056] He, F. , Su, W. , Wu, R. , Li, H. , Lou, L. , Wu, A. , Xie, L. , du, Y. , & Wu, S. (2021). The effect of cold exposure on serum cholesterol is dependent upon ApoE. Journal of Thermal Biology, 99, 102972. 34420615 10.1016/j.jtherbio.2021.102972

[phy270838-bib-0057] Hoeke, G. , Nahon, K. J. , Bakker, L. E. H. , Norkauer, S. S. C. , Dinnes, D. L. M. , Kockx, M. , Lichtenstein, L. , Drettwan, D. , Reifel‐Miller, A. , Coskun, T. , Pagel, P. , Romijn, F. P. H. T. M. , Cobbaert, C. M. , Jazet, I. M. , Martinez, L. O. , Kritharides, L. , Berbée, J. F. P. , Boon, M. R. , & Rensen, P. C. N. (2017). Short‐term cooling increases serum triglycerides and small high‐density lipoprotein levels in humans. Journal of Clinical Lipidology, 11(4), 920–928. 28625343 10.1016/j.jacl.2017.04.117

[phy270838-bib-0058] Hoi, J. K. , Lieder, B. , Liebisch, B. , Czech, C. , Hans, J. , Ley, J. P. , & Somoza, V. (2020). TRPA1 agonist Cinnamaldehyde decreases Adipogenesis in 3T3‐L1 cells more potently than the non‐agonist structural analog Cinnamyl Isobutyrate. ACS Omega, 5(51), 33305–33313. 33403292 10.1021/acsomega.0c05083 PMC7774270

[phy270838-bib-0059] Horing, M. , Brunner, S. , Scheiber, J. , Honecker, J. , Liebisch, G. , Seeliger, C. , & Schinhammer, L. (2025). Sex‐specific response of the human plasma lipidome to short‐term cold exposure. Biochimica et Biophysica Acta ‐ Molecular and Cell Biology of Lipids, 1870(1), 159567. 39366508 10.1016/j.bbalip.2024.159567

[phy270838-bib-0060] Hotamisligil, G. S. (2017). Inflammation, metaflammation and immunometabolic disorders. Nature, 542(7640), 177–185. 28179656 10.1038/nature21363

[phy270838-bib-0061] Huo, D. L. , Bao, M. H. , Cao, J. , & Zhao, Z. J. (2022). Cold exposure prevents fat accumulation in striped hamsters refed a high‐fat diet following food restriction. BMC Zoology, 7(1), 19. 37170304 10.1186/s40850-022-00122-z PMC10127302

[phy270838-bib-0062] Ikeda, K. , Kang, Q. , Yoneshiro, T. , Camporez, J. P. , Maki, H. , Homma, M. , Shinoda, K. , Chen, Y. , Lu, X. , Maretich, P. , Tajima, K. , Ajuwon, K. M. , Soga, T. , & Kajimura, S. (2017). UCP1‐independent signaling involving SERCA2b‐mediated calcium cycling regulates beige fat thermogenesis and systemic glucose homeostasis. Nature Medicine, 23(12), 1454–1465. 10.1038/nm.4429 PMC5727902 29131158

[phy270838-bib-0063] Ivanova, Y. M. , & Blondin, D. P. (2021). Examining the benefits of cold exposure as a therapeutic strategy for obesity and type 2 diabetes. Journal of Applied Physiology (1985), 130(5), 1448–1459. 10.1152/japplphysiol.00934.2020 33764169

[phy270838-bib-0064] Iwen, K. A. , Backhaus, J. , Cassens, M. , Waltl, M. , Hedesan, O. C. , Merkel, M. , Heeren, J. , Sina, C. , Rademacher, L. , Windjäger, A. , Haug, A. R. , Kiefer, F. W. , Lehnert, H. , & Schmid, S. M. (2017). Cold‐induced Brown adipose tissue activity alters plasma fatty acids and improves glucose metabolism in men. The Journal of Clinical Endocrinology and Metabolism, 102(11), 4226–4234. 28945846 10.1210/jc.2017-01250

[phy270838-bib-0065] Jacobsen, J. M. , Petersen, N. , Torz, L. , Gerstenberg, M. K. , Pedersen, K. , Østergaard, S. , Wulff, B. S. , Andersen, B. , Raun, K. , Christoffersen, B. Ø. , John, L. M. , Reitman, M. L. , & Kuhre, R. E. (2024). Housing mice near vs. below thermoneutrality affects drug‐induced weight loss but does not improve prediction of efficacy in humans. Cell Reports, 43(8), 114501. 39067024 10.1016/j.celrep.2024.114501 PMC11380917

[phy270838-bib-0066] Ji, Q. , & Salomon, A. R. (2015). Wide‐scale quantitative phosphoproteomic analysis reveals that cold treatment of T cells closely mimics soluble antibody stimulation. Journal of Proteome Research, 14(5), 2082–2089. 25839225 10.1021/pr501172u PMC4428545

[phy270838-bib-0067] Jiang, J. , Emont, M. P. , Jun, H. , Qiao, X. , Liao, J. , Kim, D. I. , & Wu, J. (2017). Cinnamaldehyde induces fat cell‐autonomous thermogenesis and metabolic reprogramming. Metabolism, 77, 58–64. 29046261 10.1016/j.metabol.2017.08.006 PMC5685898

[phy270838-bib-0068] Jurado‐Fasoli, L. , Sanchez‐Delgado, G. , di, X. , Yang, W. , Kohler, I. , Villarroya, F. , Aguilera, C. M. , Hankemeier, T. , Ruiz, J. R. , & Martinez‐Tellez, B. (2024). Cold‐induced changes in plasma signaling lipids are associated with a healthier cardiometabolic profile independently of brown adipose tissue. Cell Reports Medicine, 5(2), 101387. 38262411 10.1016/j.xcrm.2023.101387 PMC10897514

[phy270838-bib-0069] Kajimura, S. , & Spiegelman, B. M. (2020). Confounding issues in the “humanized” BAT of mice. Nature Metabolism, 2(4), 303–304. 10.1038/s42255-020-0192-y PMC7220110 32405619

[phy270838-bib-0070] Kazak, L. , Chouchani, E. T. , Jedrychowski, M. P. , Erickson, B. K. , Shinoda, K. , Cohen, P. , Vetrivelan, R. , Lu, G. Z. , Laznik‐Bogoslavski, D. , Hasenfuss, S. C. , Kajimura, S. , Gygi, S. P. , & Spiegelman, B. M. (2015). A creatine‐driven substrate cycle enhances energy expenditure and thermogenesis in beige fat. Cell, 163(3), 643–655. 26496606 10.1016/j.cell.2015.09.035 PMC4656041

[phy270838-bib-0071] Kibler, H. H. , & Johnson, H. D. (1961). Metabolic rate and aging in rats during exposure to cold. Journal of Gerontology, 16, 13–16. 13755802 10.1093/geronj/16.1.13

[phy270838-bib-0072] Kobayashi, K. , Fukuoka, T. , Obata, K. , Yamanaka, H. , Dai, Y. , Tokunaga, A. , & Noguchi, K. (2005). Distinct expression of TRPM8, TRPA1, and TRPV1 mRNAs in rat primary afferent neurons with adelta/c‐fibers and colocalization with trk receptors. The Journal of Comparative Neurology, 493(4), 596–606. 16304633 10.1002/cne.20794

[phy270838-bib-0073] Kokolus, K. M. , Capitano, M. L. , Lee, C. T. , Eng, J. W. L. , Waight, J. D. , Hylander, B. L. , Sexton, S. , Hong, C. C. , Gordon, C. J. , Abrams, S. I. , & Repasky, E. A. (2013). Baseline tumor growth and immune control in laboratory mice are significantly influenced by subthermoneutral housing temperature. Proceedings of the National Academy of Sciences of the United States of America, 110(50), 20176–20181. 24248371 10.1073/pnas.1304291110 PMC3864348

[phy270838-bib-0074] Kong, Y. , McNaboe, R. , Hossain, M. B. , Posada‐Quintero, H. F. , Diaz, K. , Chon, K. H. , & Bolkhovsky, J. (2025). Detection of cognitive performance deterioration due to cold‐air exposure in females using wearable Electrodermal activity and electrocardiogram. Biosensors‐Basel, 15(2), 78. 39996980 10.3390/bios15020078 PMC11852572

[phy270838-bib-0075] Kulterer, O. C. , Herz, C. T. , Prager, M. , Schmöltzer, C. , Langer, F. B. , Prager, G. , Marculescu, R. , Kautzky‐Willer, A. , Hacker, M. , Haug, A. R. , & Kiefer, F. W. (2022). Brown adipose tissue prevalence is lower in obesity but its metabolic activity is intact. Frontiers Endocrinology (Lausanne), 13, 858417. 10.3389/fendo.2022.858417 PMC9009254 35432192

[phy270838-bib-0076] Lauby‐Secretan, B. , Scoccianti, C. , Loomis, D. , Grosse, Y. , Bianchini, F. , Straif, K. , & International Agency for Research on Cancer Handbook Working Group . (2016). Body fatness and cancer–viewpoint of the IARC working group. The New England Journal of Medicine, 375(8), 794–798. 27557308 10.1056/NEJMsr1606602 PMC6754861

[phy270838-bib-0077] Lee, P. , Zhao, J. T. , Swarbrick, M. M. , Gracie, G. , Bova, R. , Greenfield, J. R. , Freund, J. , & Ho, K. K. Y. (2011). High prevalence of brown adipose tissue in adult humans. The Journal of Clinical Endocrinology and Metabolism, 96(8), 2450–2455. 21613352 10.1210/jc.2011-0487

[phy270838-bib-0078] Lee, Y. H. , Petkova, A. P. , Konkar, A. A. , & Granneman, J. G. (2015). Cellular origins of cold‐induced brown adipocytes in adult mice. The FASEB Journal, 29(1), 286–299. 25392270 10.1096/fj.14-263038 PMC4285542

[phy270838-bib-0079] Leiria, L. O. , Wang, C. H. , Lynes, M. D. , Yang, K. , Shamsi, F. , & Sato, M. (2019). 12‐lipoxygenase regulates cold adaptation and glucose metabolism by producing the Omega‐3 lipid 12‐HEPE from Brown fat. Cell Metabolism, 30(4), 768–783. 31353262 10.1016/j.cmet.2019.07.001 PMC6774888

[phy270838-bib-0080] Leitner, B. P. , Huang, S. , Brychta, R. J. , Duckworth, C. J. , Baskin, A. S. , McGehee, S. , Tal, I. , Dieckmann, W. , Gupta, G. , Kolodny, G. M. , Pacak, K. , Herscovitch, P. , Cypess, A. M. , & Chen, K. Y. (2017). Mapping of human brown adipose tissue in lean and obese young men. Proceedings of the National Academy of Sciences of the United States of America, 114(32), 8649–8654. 28739898 10.1073/pnas.1705287114 PMC5559032

[phy270838-bib-0081] Li, F. , Yang, W. , Jiang, H. , Guo, C. , Huang, A. J. W. , Hu, H. , & Liu, Q. (2019). TRPV1 activity and substance P release are required for corneal cold nociception. Nature Communications, 10(1), 5678. 10.1038/s41467-019-13536-0 PMC6908618 31831729

[phy270838-bib-0082] Li, F. X. , Xu, F. , Li, C. C. , Lei, L. M. , & Shan, S. K. (2024). Cold exposure alleviates T2DM through plasma‐derived extracellular vesicles. International Journal of Nanomedicine, 19, 10077–10095. 39371478 10.2147/IJN.S441847 PMC11456273

[phy270838-bib-0083] Li, X. , Ren, Y. , Chang, K. , Wu, W. , Griffiths, H. R. , Lu, S. , & Gao, D. (2023). Adipose tissue macrophages as potential targets for obesity and metabolic diseases. Frontiers in Immunology, 14, 1153915. 37153549 10.3389/fimmu.2023.1153915 PMC10154623

[phy270838-bib-0084] Lidell, M. E. , Betz, M. J. , Leinhard, O. D. , Heglind, M. , Elander, L. , Slawik, M. , Mussack, T. , Nilsson, D. , Romu, T. , Nuutila, P. , Virtanen, K. A. , Beuschlein, F. , Persson, A. , Borga, M. , & Enerbäck, S. (2013). Evidence for two types of brown adipose tissue in humans. Nature Medicine, 19(5), 631–634. 10.1038/nm.3017 23603813

[phy270838-bib-0085] Liskiewicz, D. , Zhang, Q. , Barthem, C. S. , Jastroch, M. , Liskiewicz, A. , Khajavi, N. , Grandl, G. , Coupland, C. , Kleinert, M. , Garcia‐Caceres, C. , Novikoff, A. , Maity, G. , Boehm, U. , Tschöp, M. H. , & Müller, T. D. (2023). Neuronal loss of TRPM8 leads to obesity and glucose intolerance in male mice. Molecular Metabolism, 72, 101714. 36966947 10.1016/j.molmet.2023.101714 PMC10106965

[phy270838-bib-0086] Liu, Y. , Mikrani, R. , He, Y. , Faran Ashraf Baig, M. M. , Abbas, M. , Naveed, M. , Tang, M. , Zhang, Q. , Li, C. , & Zhou, X. (2020). TRPM8 channels: A review of distribution and clinical role. European Journal of Pharmacology, 882, 173312. 32610057 10.1016/j.ejphar.2020.173312

[phy270838-bib-0087] Long, J. Z. , Svensson, K. J. , Tsai, L. , Zeng, X. , Roh, H. C. , Kong, X. , Rao, R. R. , Lou, J. , Lokurkar, I. , Baur, W. , Castellot, J. J., Jr. , Rosen, E. D. , & Spiegelman, B. M. (2014). A smooth muscle‐like origin for beige adipocytes. Cell Metabolism, 19(5), 810–820. 24709624 10.1016/j.cmet.2014.03.025 PMC4052772

[phy270838-bib-0088] Lynes, M. D. , Leiria, L. O. , Lundh, M. , Bartelt, A. , Shamsi, F. , Huang, T. L. , Takahashi, H. , Hirshman, M. F. , Schlein, C. , Lee, A. , Baer, L. A. , May, F. J. , Gao, F. , Narain, N. R. , Chen, E. Y. , Kiebish, M. A. , Cypess, A. M. , Blüher, M. , Goodyear, L. J. , … Tseng, Y. H. (2017). The cold‐induced lipokine 12,13‐diHOME promotes fatty acid transport into brown adipose tissue. Nature Medicine, 23(5), 631–637. 10.1038/nm.4297 PMC5699924 28346411

[phy270838-bib-0089] Ma, S. , Yu, H. , Zhao, Z. , Luo, Z. , Chen, J. , Ni, Y. , Jin, R. , Ma, L. , Wang, P. , Zhu, Z. , Li, L. , Zhong, J. , Liu, D. , Nilius, B. , & Zhu, Z. (2012). Activation of the cold‐sensing TRPM8 channel triggers UCP1‐dependent thermogenesis and prevents obesity. Journal of Molecular Cell Biology, 4(2), 88–96. 22241835 10.1093/jmcb/mjs001

[phy270838-bib-0090] Maiello, M. , Boeri, D. , Bonadonna, R. , Odetti, P. , Saccarello, A. , & Adezati, L. (1988). Platelet and clotting activities after cold stress in diabetic patients. Thrombosis Research, 50(6), 885–894. 2970690 10.1016/0049-3848(88)90348-9

[phy270838-bib-0091] Makarova, O. V. , Trunova, G. V. , Diatroptov, M. E. , Serebryakov, S. N. , Kondashevskaya, M. V. , & Malaitsev, V. V. (2005). Comparative characterization of cytokine production by concanavalin A‐activated splenocytes from BALB/c and C57BL/6 mice after cold exposure. Bulletin of Experimental Biology and Medicine, 139(2), 220–222. 16027812 10.1007/s10517-005-0253-y

[phy270838-bib-0092] Marini, M. , Titiz, M. , Souza Monteiro de Araújo, D. , Geppetti, P. , Nassini, R. , & de Logu, F. (2023). TRP channels in cancer: Signaling mechanisms and translational approaches. Biomolecules, 13(10), 1557. 37892239 10.3390/biom13101557 PMC10605459

[phy270838-bib-0093] McInnis, K. , Haman, F. , & Doucet, E. (2020). Humans in the cold: Regulating energy balance. Obesity Reviews, 21(3), e12978. 31863637 10.1111/obr.12978

[phy270838-bib-0094] McInnis, K. , Larocque, A. , Beauregard, N. , Hintze, L. J. , Finlayson, G. , Haman, F. , & Doucet, É. (2025). Energy intake and energy expenditure are minimally impacted by acute cold exposure in individuals living with obesity. International Journal of Obesity, 49(8), 1654–1661. 40410569 10.1038/s41366-025-01809-2

[phy270838-bib-0095] McKie, G. L. , Medak, K. D. , Shamshoum, H. , & Wright, D. C. (2022). Topical application of the pharmacological cold mimetic menthol stimulates brown adipose tissue thermogenesis through a TRPM8, UCP1, and norepinephrine dependent mechanism in mice housed at thermoneutrality. The FASEB Journal, 36(3), e22205. 35157333 10.1096/fj.202101905RR

[phy270838-bib-0096] McKie, G. L. , Shamshoum, H. , Hunt, K. L. , Thorpe, H. H. A. , Dibe, H. A. , Khokhar, J. Y. , Doucette, C. A. , & Wright, D. C. (2022). Intermittent cold exposure improves glucose homeostasis despite exacerbating diet‐induced obesity in mice housed at thermoneutrality. The Journal of Physiology, 600(4), 829–845. 34192813 10.1113/JP281774

[phy270838-bib-0097] Mengel, L. A. , Nemati Moud, B. , Seidl, H. , Mesas‐Fernández, A. , Seeliger, C. , Brandl, B. , Skurk, T. , Holzapfel, C. , Claussnitzer, M. , & Hauner, H. (2022). Effect of BMI on the Thermogenic response to cold exposure and associated changes in metabolism and Browning markers in adult humans. Obesity Facts, 15(3), 405–415. 35081533 10.1159/000522218 PMC9210029

[phy270838-bib-0098] Mooventhan, A. , Chaudhari, S. S. , & Venugopal, V. (2020). Effect of cold hip bath on blood glucose levels in patients with type 2 diabetes mellitus: A pilot study. Diabetes & Metabolism, 46(5), 411–412. 31018165 10.1016/j.diabet.2019.04.003

[phy270838-bib-0099] Mouisel, E. , Bodon, A. , Noll, C. , Cassant‐Sourdy, S. , Marques, M. A. , Flores‐Flores, R. , Riant, E. , Bergoglio, C. , Vezin, P. , & Caspar‐Bauguil, S. (2025). Cold‐induced thermogenesis requires neutral‐lipase‐mediated intracellular lipolysis in brown adipocytes. Cell Metabolism, 37(2), 429–440. 39566492 10.1016/j.cmet.2024.10.018

[phy270838-bib-0100] Nguyen, M. Q. , von Buchholtz, L. J. , Reker, A. N. , Ryba, N. J. P. , & Davidson, S. (2021). Single‐nucleus transcriptomic analysis of human dorsal root ganglion neurons. eLife, 10, 10. 10.7554/eLife.71752 PMC8626086 34825887

[phy270838-bib-0101] Nikami, H. , Shimizu, Y. , Endoh, D. , Yano, H. , & Saito, M. (1992). Cold exposure increases glucose utilization and glucose transporter expression in brown adipose tissue. Biochemical and Biophysical Research Communications, 185(3), 1078–1082. 1378263 10.1016/0006-291x(92)91736-a

[phy270838-bib-0102] Nisoli, E. , & Cinti, S. (2024). What defines a cell type? Perspectives from adipocyte biology. International Journal of Obesity, 49, 751–754. 39627607 10.1038/s41366-024-01696-z PMC12095039

[phy270838-bib-0103] Nunes, J. R. C. , Smith, T. K. T. , Ghorbani, P. , O'Dwyer, C. , Trzaskalski, N. A. , Dergham, H. , Pember, C. , Kilgour, M. K. , Mulvihill, E. E. , & Fullerton, M. D. (2023). Thermoneutral housing does not accelerate metabolic dysfunction‐associated fatty liver disease in male or female C57Bl/6J mice fed a Western diet. American Journal of Physiology. Endocrinology and Metabolism, 325(1), E10–E20. 37196059 10.1152/ajpendo.00124.2023

[phy270838-bib-0104] Oliveira, R. L. , Ueno, M. , de Souza, C. T. , Pereira‐da‐Silva, M. , Gasparetti, A. L. , & Bezzera, R. M. (2004). Cold‐induced PGC‐1alpha expression modulates muscle glucose uptake through an insulin receptor/Akt‐independent, AMPK‐dependent pathway. American Journal of Physiology. Endocrinology and Metabolism, 287(4), E686–E695. 15165993 10.1152/ajpendo.00103.2004

[phy270838-bib-0105] Ong, F. J. , Ahmed, B. A. , Oreskovich, S. M. , Blondin, D. P. , Haq, T. , Konyer, N. B. , Noseworthy, M. D. , Haman, F. , Carpentier, A. C. , Morrison, K. M. , & Steinberg, G. R. (2018). Recent advances in the detection of brown adipose tissue in adult humans: A review. Clinical Science (London, England), 132(10), 1039–1054. 10.1042/CS20170276 29802209

[phy270838-bib-0106] Orava, J. , Nuutila, P. , Lidell, M. E. , Oikonen, V. , Noponen, T. , Viljanen, T. , Scheinin, M. , Taittonen, M. , Niemi, T. , Enerbäck, S. , & Virtanen, K. A. (2011). Different metabolic responses of human brown adipose tissue to activation by cold and insulin. Cell Metabolism, 14(2), 272–279. 21803297 10.1016/j.cmet.2011.06.012

[phy270838-bib-0107] Ouellet, V. , Labbé, S. M. , Blondin, D. P. , Phoenix, S. , Guérin, B. , Haman, F. , Turcotte, E. E. , Richard, D. , & Carpentier, A. C. (2012). Brown adipose tissue oxidative metabolism contributes to energy expenditure during acute cold exposure in humans. The Journal of Clinical Investigation, 122(2), 545–552. 22269323 10.1172/JCI60433 PMC3266793

[phy270838-bib-0108] Park, C. H. , Park, M. , Kelly, M. E. , Cheng, H. , Lee, S. R. , Jang, C. , & Chang, J. S. (2025). Cold‐inducible GOT1 activates the malate‐aspartate shuttle in brown adipose tissue to support fuel preference for fatty acids. Cell Reports, 44(7), 115888. 40540398 10.1016/j.celrep.2025.115888 PMC12294511

[phy270838-bib-0109] Park, G. , Haley, J. A. , le, J. , Jung, S. M. , Fitzgibbons, T. P. , Korobkina, E. D. , Li, H. , Fluharty, S. M. , Chen, Q. , Spinelli, J. B. , Trivedi, C. M. , Jang, C. , & Guertin, D. A. (2023). Quantitative analysis of metabolic fluxes in brown fat and skeletal muscle during thermogenesis. Nature Metabolism, 5(7), 1204–1220. 10.1038/s42255-023-00825-8 PMC10696589 37337122

[phy270838-bib-0110] Paulus, A. , Drude, N. , van Marken Lichtenbelt, W. , Mottaghy, F. M. , & Bauwens, M. (2020). Brown adipose tissue uptake of triglyceride‐rich lipoprotein‐derived fatty acids in diabetic or obese mice under different temperature conditions. EJNMMI Research, 10(1), 127. 33085016 10.1186/s13550-020-00701-6 PMC7578207

[phy270838-bib-0111] Peier, A. M. , Moqrich, A. , Hergarden, A. C. , Reeve, A. J. , Andersson, D. A. , Story, G. M. , Earley, T. J. , Dragoni, I. , McIntyre, P. , Bevan, S. , & Patapoutian, A. (2002). A TRP channel that senses cold stimuli and menthol. Cell, 108(5), 705–715. 11893340 10.1016/s0092-8674(02)00652-9

[phy270838-bib-0112] Pernes, G. , Morgan, P. K. , Huynh, K. , Mellett, N. A. , Meikle, P. J. , Murphy, A. J. , Henstridge, D. C. , & Lancaster, G. I. (2021). Characterization of the circulating and tissue‐specific alterations to the lipidome in response to moderate and major cold stress in mice. American Journal of Physiology. Regulatory, Integrative and Comparative Physiology, 320(2), R95–R104. 33175588 10.1152/ajpregu.00112.2020

[phy270838-bib-0113] Peyronnel, C. , Totoson, P. , Petitcolin, V. , Bonnefoy, F. , Guillot, X. , Saas, P. , Verhoeven, F. , Martin, H. , & Demougeot, C. (2022). Effects of local cryotherapy on systemic endothelial activation, dysfunction, and vascular inflammation in adjuvant‐induced arthritis (AIA) rats. Arthritis Research & Therapy, 24(1), 97. 35488311 10.1186/s13075-022-02774-1 PMC9052534

[phy270838-bib-0114] Racinais, S. , Dablainville, V. , Rousse, Y. , Ihsan, M. , Grant, M. E. , Schobersberger, W. , Budgett, R. , & Engebretsen, L. (2024). Cryotherapy for treating soft tissue injuries in sport medicine: A critical review. British Journal of Sports Medicine, 58(20), 1215–1223. 39237265 10.1136/bjsports-2024-108304

[phy270838-bib-0115] Rahbani, J. F. , Bunk, J. , Lagarde, D. , Samborska, B. , Roesler, A. , Xiao, H. , Shaw, A. , Kaiser, Z. , Braun, J. L. , Geromella, M. S. , Fajardo, V. A. , Koza, R. A. , & Kazak, L. (2024). Parallel control of cold‐triggered adipocyte thermogenesis by UCP1 and CKB. Cell Metabolism, 36(3), 526–540. 38272036 10.1016/j.cmet.2024.01.001

[phy270838-bib-0116] Raun, S. H. , Braun, J. L. , Karavaeva, I. , Henriquez‐Olguín, C. , Ali, M. S. , Møller, L. L. V. , Gerhart‐Hines, Z. , Fajardo, V. A. , Richter, E. A. , & Sylow, L. (2024). Mild cold stress at ambient temperature elevates muscle calcium cycling and exercise adaptations in obese female mice. Endocrinology, 165(10), bqae102. 39136248 10.1210/endocr/bqae102

[phy270838-bib-0117] Ravussin, Y. , Xiao, C. , Gavrilova, O. , & Reitman, M. L. (2014). Effect of intermittent cold exposure on brown fat activation, obesity, and energy homeostasis in mice. PLoS One, 9(1), e85876. 24465761 10.1371/journal.pone.0085876 PMC3895006

[phy270838-bib-0118] Reimundez, A. , Fernández‐Peña, C. , García, G. , Fernández, R. , Ordás, P. , Gallego, R. , Pardo‐Vazquez, J. L. , Arce, V. , Viana, F. , & Señarís, R. (2018). Deletion of the cold Thermoreceptor TRPM8 increases heat loss and food intake leading to reduced body temperature and obesity in mice. Journal of Neuroscience, 38(15), 3643–3656. 29530988 10.1523/JNEUROSCI.3002-17.2018 PMC6705917

[phy270838-bib-0119] Remie, C. M. E. , Moonen, M. P. B. , Roumans, K. H. M. , Nascimento, E. B. M. , Gemmink, A. , Havekes, B. , Schaart, G. , Kornips, E. , Joris, P. J. , Schrauwen‐Hinderling, V. B. , Hoeks, J. , Kersten, S. , Hesselink, M. K. C. , Phielix, E. , Lichtenbelt, W. D. M. , & Schrauwen, P. (2021). Metabolic responses to mild cold acclimation in type 2 diabetes patients. Nature Communications, 12(1), 1516. 10.1038/s41467-021-21813-0 PMC7943816 33750795

[phy270838-bib-0120] Reynés, B. , van Schothorst, E. M. , García‐Ruiz, E. , Keijer, J. , Palou, A. , & Oliver, P. (2017). Cold exposure down‐regulates immune response pathways in ferret aortic perivascular adipose tissue. Thrombosis and Haemostasis, 117(5), 981–991. 28251235 10.1160/TH16-12-0931

[phy270838-bib-0121] Saari, T. J. , Raiko, J. , U‐Din, M. , Niemi, T. , Taittonen, M. , Laine, J. , Savisto, N. , Haaparanta‐Solin, M. , Nuutila, P. , & Virtanen, K. A. (2020). Basal and cold‐induced fatty acid uptake of human brown adipose tissue is impaired in obesity. Scientific Reports, 10(1), 14373. 32873825 10.1038/s41598-020-71197-2 PMC7463032

[phy270838-bib-0122] Saito, M. , Okamatsu‐Ogura, Y. , Matsushita, M. , Watanabe, K. , Yoneshiro, T. , Nio‐Kobayashi, J. , Iwanaga, T. , Miyagawa, M. , Kameya, T. , Nakada, K. , Kawai, Y. , & Tsujisaki, M. (2009). High incidence of metabolically active brown adipose tissue in healthy adult humans: Effects of cold exposure and adiposity. Diabetes, 58(7), 1526–1531. 19401428 10.2337/db09-0530 PMC2699872

[phy270838-bib-0123] Sakers, A. , de Siqueira, M. K. , Seale, P. , & Villanueva, C. J. (2022). Adipose‐tissue plasticity in health and disease. Cell, 185(3), 419–446. 35120662 10.1016/j.cell.2021.12.016 PMC11152570

[phy270838-bib-0124] Sanders, O. D. , Rajagopal, J. A. , & Rajagopal, L. (2021). Menthol to induce non‐shivering thermogenesis via TRPM8/PKA signaling for treatment of obesity. Journal of Obesity & Metabolic Syndrome, 30(1), 4–11. 33071240 10.7570/jomes20038 PMC8017329

[phy270838-bib-0125] Sankina, P. , Lal, R. , Khare, P. , von Hörsten, S. , Fester, L. , Aggarwal, V. , Zimmermann, K. , & Bishnoi, M. (2024). Topical menthol, a pharmacological cold mimic, induces cold sensitivity, adaptive thermogenesis and brown adipose tissue activation in mice. Diabetes, Obesity & Metabolism, 26, 4329–4345. 10.1111/dom.15781 39044311

[phy270838-bib-0126] Schutz, M. , Oertel, B. G. , Heimann, D. , Doehring, A. , Walter, C. , Dimova, V. , Geisslinger, G. , & Lötsch, J. (2014). Consequences of a human TRPA1 genetic variant on the perception of nociceptive and olfactory stimuli. PLoS One, 9(4), e95592. 24752136 10.1371/journal.pone.0095592 PMC4005389

[phy270838-bib-0127] Seale, P. , Bjork, B. , Yang, W. , Kajimura, S. , Chin, S. , Kuang, S. , Scimè, A. , Devarakonda, S. , Conroe, H. M. , Erdjument‐Bromage, H. , Tempst, P. , Rudnicki, M. A. , Beier, D. R. , & Spiegelman, B. M. (2008). PRDM16 controls a brown fat/skeletal muscle switch. Nature, 454(7207), 961–967. 18719582 10.1038/nature07182 PMC2583329

[phy270838-bib-0128] Seki, T. , Yang, Y. , Sun, X. , Lim, S. , Xie, S. , Guo, Z. , Xiong, W. , Kuroda, M. , Sakaue, H. , Hosaka, K. , Jing, X. , Yoshihara, M. , Qu, L. , Li, X. , Chen, Y. , & Cao, Y. (2022). Brown‐fat‐mediated tumour suppression by cold‐altered global metabolism. Nature, 608(7922), 421–428. 35922508 10.1038/s41586-022-05030-3 PMC9365697

[phy270838-bib-0129] Sellers, A. J. , Pallubinsky, H. , Rense, P. , Bijnens, W. , van de Weijer, T. , Moonen‐Kornips, E. , Schrauwen, P. , & van Marken Lichtenbelt, W. D. (2021). The effect of cold exposure with shivering on glucose tolerance in healthy men. Journal of Applied Physiology, 130(1), 193–205. 33090911 10.1152/japplphysiol.00642.2020

[phy270838-bib-0130] Sellers, A. J. , van Beek, S. M. M. , Hashim, D. , Baak, R. , Pallubinsky, H. , Moonen‐Kornips, E. , Schaart, G. , Gemmink, A. , Jörgensen, J. A. , van de Weijer, T. , Kalkhoven, E. , Hooiveld, G. J. , Kersten, S. , Hesselink, M. K. C. , Schrauwen, P. , Hoeks, J. , & van Marken Lichtenbelt, W. D. (2024). Cold acclimation with shivering improves metabolic health in adults with overweight or obesity. Nature Metabolism, 6(12), 2246–2253. 10.1038/s42255-024-01172-y 39643644

[phy270838-bib-0131] Senn, J. R. , Maushart, C. I. , Gashi, G. , Michel, R. , Lalive d'Epinay, M. , Vogt, R. , Becker, A. S. , Müller, J. , Baláz, M. , Wolfrum, C. , Burger, I. A. , & Betz, M. J. (2018). Outdoor temperature influences cold induced thermogenesis in humans. Frontiers in Physiology, 9, 1184. 30190681 10.3389/fphys.2018.01184 PMC6115528

[phy270838-bib-0132] Shinoda, K. , Luijten, I. H. N. , Hasegawa, Y. , Hong, H. , Sonne, S. B. , Kim, M. , Xue, R. , Chondronikola, M. , Cypess, A. M. , Tseng, Y. H. , Nedergaard, J. , Sidossis, L. S. , & Kajimura, S. (2015). Genetic and functional characterization of clonally derived adult human brown adipocytes. Nature Medicine, 21(4), 389–394. 10.1038/nm.3819 PMC4427356 25774848

[phy270838-bib-0133] Singh, A. K. , Aryal, B. , Chaube, B. , Rotllan, N. , Varela, L. , Horvath, T. L. , Suárez, Y. , & Fernández‐Hernando, C. (2018). Brown adipose tissue derived ANGPTL4 controls glucose and lipid metabolism and regulates thermogenesis. Molecular Metabolism, 11, 59–69. 29627378 10.1016/j.molmet.2018.03.011 PMC6001401

[phy270838-bib-0134] Singh, R. , Barrios, A. , Dirakvand, G. , & Pervin, S. (2021). Human Brown adipose tissue and metabolic health: Potential for therapeutic avenues. Cells, 10(11), 3030. 34831253 10.3390/cells10113030 PMC8616549

[phy270838-bib-0135] Smith, O. L. , & Davidson, S. B. (1982). Shivering thermogenesis and glucose uptake by muscles of normal or diabetic rats. The American Journal of Physiology, 242(1), R109–R115. 7036756 10.1152/ajpregu.1982.242.1.R109

[phy270838-bib-0136] Song, A. , Dai, W. , Jang, M. J. , Medrano, L. , Li, Z. , Zhao, H. , Shao, M. , Tan, J. , Li, A. , Ning, T. , Miller, M. M. , Armstrong, B. , Huss, J. M. , Zhu, Y. , Liu, Y. , Gradinaru, V. , Wu, X. , Jiang, L. , Scherer, P. E. , & Wang, Q. A. (2020). Low‐ and high‐thermogenic brown adipocyte subpopulations coexist in murine adipose tissue. The Journal of Clinical Investigation, 130(1), 247–257. 31573981 10.1172/JCI129167 PMC6934193

[phy270838-bib-0137] Spiljar, M. , Steinbach, K. , Rigo, D. , Suárez‐Zamorano, N. , Wagner, I. , Hadadi, N. , Vincenti, I. , Page, N. , Klimek, B. , Rochat, M. A. , Kreutzfeldt, M. , Chevalier, C. , Stojanović, O. , Bejuy, O. , Colin, D. , Mack, M. , Cansever, D. , Greter, M. , Merkler, D. , & Trajkovski, M. (2021). Cold exposure protects from neuroinflammation through immunologic reprogramming. Cell Metabolism, 33(11), 2231–2246. 34687652 10.1016/j.cmet.2021.10.002 PMC8570411

[phy270838-bib-0138] Stahl, V. , Maier, F. , Freitag, M. T. , Floca, R. O. , Berger, M. C. , Umathum, R. , Berriel Diaz, M. , Herzig, S. , Weber, M. A. , Dimitrakopoulou‐Strauss, A. , Rink, K. , Bachert, P. , Ladd, M. E. , & Nagel, A. M. (2017). In vivo assessment of cold stimulation effects on the fat fraction of brown adipose tissue using DIXON MRI. Journal of Magnetic Resonance Imaging, 45(2), 369–380. 27421080 10.1002/jmri.25364

[phy270838-bib-0139] Steiner, G. G. , Steiner, G. G. , & Steiner, G. G. (2002). Cancer incidence rates and environmental factors: An ecological study. Journal of Environmental Pathology, Toxicology and Oncology, 21(3), 205–212. 12435073

[phy270838-bib-0140] Story, G. M. , Peier, A. M. , Reeve, A. J. , Eid, S. R. , Mosbacher, J. , Hricik, T. R. , Earley, T. J. , Hergarden, A. C. , Andersson, D. A. , Hwang, S. W. , McIntyre, P. , Jegla, T. , Bevan, S. , & Patapoutian, A. (2003). ANKTM1, a TRP‐like channel expressed in nociceptive neurons, is activated by cold temperatures. Cell, 112(6), 819–829. 12654248 10.1016/s0092-8674(03)00158-2

[phy270838-bib-0141] Straat, M. E. , Jurado‐Fasoli, L. , Ying, Z. , Nahon, K. J. , Janssen, L. G. M. , Boon, M. R. , Grabner, G. F. , Kooijman, S. , Zimmermann, R. , Giera, M. , Rensen, P. C. N. , & Martinez‐Tellez, B. (2022). Cold exposure induces dynamic changes in circulating triacylglycerol species, which is dependent on intracellular lipolysis: A randomized cross‐over trial. eBioMedicine, 86, 104349. 36371986 10.1016/j.ebiom.2022.104349 PMC9663865

[phy270838-bib-0142] Sugimoto, S. , Mena, H. A. , Sansbury, B. E. , Kobayashi, S. , Tsuji, T. , Wang, C. H. , Yin, X. , Huang, T. L. , Kusuyama, J. , Kodani, S. D. , Darcy, J. , Profeta, G. , Pereira, N. , Tanzi, R. E. , Zhang, C. , Serwold, T. , Kokkotou, E. , Goodyear, L. J. , Cypess, A. M. , … Tseng, Y. H. (2022). Brown adipose tissue‐derived MaR2 contributes to cold‐induced resolution of inflammation. Nature Metabolism, 4(6), 775–790. 10.1038/s42255-022-00590-0 PMC9792164 35760872

[phy270838-bib-0143] Tashani, O. A. , Astita, R. , Sharp, D. , & Johnson, M. I. (2017). Body mass index and distribution of body fat can influence sensory detection and pain sensitivity. European Journal of Pain, 21(7), 1186–1196. 28263427 10.1002/ejp.1019

[phy270838-bib-0144] Tian, L. , Qiu, H. , Sun, S. , & Lin, H. (2016). Emergency cardiovascular hospitalization risk attributable to cold temperatures in Hong Kong. Circulation. Cardiovascular Quality and Outcomes, 9(2), 135–142. 26933049 10.1161/CIRCOUTCOMES.115.002410

[phy270838-bib-0145] Townsend, L. K. , Wang, D. , Wright, D. C. , & Blondin, D. P. (2023). Skeletal muscle, not adipose tissue, mediates cold‐induced metabolic benefits. Nature Metabolism, 5(7), 1074–1077. 10.1038/s42255-023-00837-4 37365377

[phy270838-bib-0146] Unlu, Y. , Piaggi, P. , Stinson, E. J. , de Baca, T. C. , Rodzevik, T. L. , Walter, M. , Fry, H. , Krakoff, J. , & Chang, D. C. (2025). Cold induces increased ad libitum energy intake independent of changes in energy expenditure: A controlled crossover trial in adults. The American Journal of Clinical Nutrition, 121(2), 293–303. 39675563 10.1016/j.ajcnut.2024.12.013 PMC11863325

[phy270838-bib-0147] Vaanholt, L. M. , Daan, S. , Schubert, K. A. , & Visser, G. H. (2009). Metabolism and aging: Effects of cold exposure on metabolic rate, body composition, and longevity in mice. Physiological and Biochemical Zoology, 82(4), 314–324. 19115965 10.1086/589727

[phy270838-bib-0148] Vallerand, A. L. , Lupien, J. , & Bukowiecki, L. J. (1986). Cold exposure reverses the diabetogenic effects of high‐fat feeding. Diabetes, 35(3), 329–334. 3005094 10.2337/diab.35.3.329

[phy270838-bib-0149] van Beek, S. , Hashim, D. , Bengtsson, T. , & Hoeks, J. (2023). Physiological and molecular mechanisms of cold‐induced improvements in glucose homeostasis in humans beyond brown adipose tissue. International Journal of Obesity, 47(5), 338–347. 36774412 10.1038/s41366-023-01270-z

[phy270838-bib-0150] van der Stelt, I. , Hoevenaars, F. , Široká, J. , De Ronde, L. , Friedecký, D. , & Keijer, J. (2017). Metabolic response of visceral white adipose tissue of obese mice exposed for 5 days to human room temperature compared to mouse Thermoneutrality. Frontiers in Physiology, 8, 179. 28386236 10.3389/fphys.2017.00179 PMC5362617

[phy270838-bib-0151] van Marken Lichtenbelt, W. D. , Vanhommerig, J. W. , Smulders, N. M. , Drossaerts, J. M. A. F. L. , Kemerink, G. J. , Bouvy, N. D. , Schrauwen, P. , & Teule, G. J. J. (2009). Cold‐activated brown adipose tissue in healthy men. The New England Journal of Medicine, 360(15), 1500–1508. 19357405 10.1056/NEJMoa0808718

[phy270838-bib-0152] Vargas‐Castillo, A. , Sun, Y. , Smythers, A. L. , Grauvogel, L. , Dumesic, P. A. , Emont, M. P. , Tsai, L. T. , Rosen, E. D. , Zammit, N. W. , Shaffer, S. M. , Ordonez, M. , Chouchani, E. T. , Gygi, S. P. , Wang, T. , Sharma, A. K. , Balaz, M. , Wolfrum, C. , & Spiegelman, B. M. (2024). Development of a functional beige fat cell line uncovers independent subclasses of cells expressing UCP1 and the futile creatine cycle. Cell Metabolism, 36(9), 2146–2155. 39084217 10.1016/j.cmet.2024.07.002 PMC12005060

[phy270838-bib-0153] Versteeg, N. , Clijsen, R. , & Hohenauer, E. (2023). Effects of 3‐week repeated cold water immersion on leukocyte counts and cardiovascular factors: An exploratory study. Frontiers in Physiology, 14, 1197585. 37711459 10.3389/fphys.2023.1197585 PMC10497764

[phy270838-bib-0154] Virtanen, K. A. , Lidell, M. E. , Orava, J. , Heglind, M. , Westergren, R. , Niemi, T. , Taittonen, M. , Laine, J. , Savisto, N. J. , Enerbäck, S. , & Nuutila, P. (2009). Functional brown adipose tissue in healthy adults. The New England Journal of Medicine, 360(15), 1518–1525. 19357407 10.1056/NEJMoa0808949

[phy270838-bib-0155] Wang, D. , Cheng, X. , Fang, H. , Ren, Y. , Li, X. , Ren, W. , Xue, B. , & Yang, C. (2020). Effect of cold stress on ovarian & uterine microcirculation in rats and the role of endothelin system. Reproductive Biology and Endocrinology, 18(1), 29. 32290862 10.1186/s12958-020-00584-1 PMC7155299

[phy270838-bib-0156] Wang, T. , Sharma, A. K. , Wu, C. , Maushart, C. I. , Ghosh, A. , Yang, W. , Stefanicka, P. , Kovanicova, Z. , Ukropec, J. , Zhang, J. , Arnold, M. , Klug, M. , De Bock, K. , Schneider, U. , Popescu, C. , Zheng, B. , Ding, L. , Long, F. , Dewal, R. S. , … Wolfrum, C. (2024). Single‐nucleus transcriptomics identifies separate classes of UCP1 and futile cycle adipocytes. Cell Metabolism, 36(9), 2130–2145. 39084216 10.1016/j.cmet.2024.07.005

[phy270838-bib-0157] Wang, T. Y. , Liu, C. , Wang, A. , & Sun, Q. (2015). Intermittent cold exposure improves glucose homeostasis associated with brown and white adipose tissues in mice. Life Sciences, 139, 153–159. 26281919 10.1016/j.lfs.2015.07.030 PMC4598301

[phy270838-bib-0158] Wang, X. , Che, H. , Zhang, W. , Wang, J. , Ke, T. , Cao, R. , Meng, S. , Li, D. , Weiming, O. , Chen, J. , & Luo, W. (2015). Effects of mild chronic intermittent cold exposure on rat organs. International Journal of Biological Sciences, 11(10), 1171–1180. 26327811 10.7150/ijbs.12161 PMC4551753

[phy270838-bib-0159] Weng, X. , Wang, C. H. A. O. G. E. , Yuan, Y. U. , Wang, Z. H. E. N. H. U. A. N. , Kuang, J. U. J. I. A. O. , Yan, X. U. , & Chen, H. A. O. (2023). Effect of cold exposure and exercise on insulin sensitivity and serum free fatty acids in obese rats. Medicine and Science in Sports and Exercise, 55(8), 1409–1415. 36924336 10.1249/MSS.0000000000003173

[phy270838-bib-0160] Wu, J. , Boström, P. , Sparks, L. M. , Ye, L. , Choi, J. H. , Giang, A. H. , Khandekar, M. , Virtanen, K. A. , Nuutila, P. , Schaart, G. , Huang, K. , Tu, H. , van Marken Lichtenbelt, W. D. , Hoeks, J. , Enerbäck, S. , Schrauwen, P. , & Spiegelman, B. M. (2012). Beige adipocytes are a distinct type of thermogenic fat cell in mouse and human. Cell, 150(2), 366–376. 22796012 10.1016/j.cell.2012.05.016 PMC3402601

[phy270838-bib-0161] Xu, Y. , Yang, Z. , & Su, C. (1992). Enhancement of cellular immune function during cold adaptation of BALB/c inbred mice. Cryobiology, 29(3), 422–427. 1499325 10.1016/0011-2240(92)90044-3

[phy270838-bib-0162] Xu, Z. , You, W. , Zhou, Y. , Chen, W. , Wang, Y. , & Shan, T. (2019). Cold‐induced lipid dynamics and transcriptional programs in white adipose tissue. BMC Biology, 17(1), 74. 31530289 10.1186/s12915-019-0693-x PMC6749700

[phy270838-bib-0163] Yahata, T. , & Kuroshima, A. (1989). Metabolic cold acclimation after repetitive intermittent cold exposure in rat. The Japanese Journal of Physiology, 39(2), 215–228. 2761120 10.2170/jjphysiol.39.215

[phy270838-bib-0164] Ye, J. , Wang, H. , Zheng, J. , Ning, S. , Zhu, D. , Shi, J. , & Shi, R. (2025). Cold exposure therapy enhances single‐atom Nanozyme‐mediated cancer vaccine therapy. ACS Applied Materials & Interfaces, 17(8), 11752–11763. 39945542 10.1021/acsami.4c20487

[phy270838-bib-0165] Yoneshiro, T. , Aita, S. , Matsushita, M. , Kayahara, T. , Kameya, T. , Kawai, Y. , Iwanaga, T. , & Saito, M. (2013). Recruited brown adipose tissue as an antiobesity agent in humans. The Journal of Clinical Investigation, 123(8), 3404–3408. 23867622 10.1172/JCI67803 PMC3726164

[phy270838-bib-0166] Yoneshiro, T. , & Saito, M. (2015). Activation and recruitment of brown adipose tissue as anti‐obesity regimens in humans. Annals of Medicine, 47(2), 133–141. 24901355 10.3109/07853890.2014.911595

[phy270838-bib-0167] Yoo, H. S. , Qiao, L. , Bosco, C. , Leong, L. H. , Lytle, N. , Feng, G. S. , Chi, N. W. , & Shao, J. (2014). Intermittent cold exposure enhances fat accumulation in mice. PLoS One, 9(5), e96432. 24789228 10.1371/journal.pone.0096432 PMC4008632

[phy270838-bib-0168] Yoshimoto, M. , Yagi, K. , Ikegame, S. , & Miki, K. (2025). Progressive increase in renal sympathetic nerve activity induced by cold exposure. Hypertension, 82(4), 615–623. 39840442 10.1161/HYPERTENSIONAHA.124.23499

[phy270838-bib-0169] Zhang, L. , An, G. , Wu, S. , Wang, J. , Yang, D. , Zhang, Y. , & Li, X. (2021). Long‐term intermittent cold exposure affects peri‐ovarian adipose tissue and ovarian microenvironment in rats. Journal of Ovarian Research, 14(1), 107. 34419111 10.1186/s13048-021-00851-8 PMC8379824

[phy270838-bib-0170] Zhao, Z. , Yang, R. , Li, M. , Bao, M. , Huo, D. , Cao, J. , & Speakman, J. R. (2022). Effects of ambient temperatures between 5 and 35 degrees C on energy balance, body mass and body composition in mice. Molecular Metabolism, 64, 101551. 35870706 10.1016/j.molmet.2022.101551 PMC9382332

[phy270838-bib-0171] Zhu, P. , Zhang, Z. H. , Huang, X. F. , Shi, Y. C. , Khandekar, N. , Yang, H. Q. , Liang, S. Y. , Song, Z. Y. , & Lin, S. (2018). Cold exposure promotes obesity and impairs glucose homeostasis in mice subjected to a high‐fat diet. Molecular Medicine Reports, 18(4), 3923–3931. 30106124 10.3892/mmr.2018.9382 PMC6131648

[phy270838-bib-0172] Zhu, X. , Bührer, C. , & Wellmann, S. (2016). Cold‐inducible proteins CIRP and RBM3, a unique couple with activities far beyond the cold. Cellular and Molecular Life Sciences, 73(20), 3839–3859. 27147467 10.1007/s00018-016-2253-7 PMC5021741

[phy270838-bib-0173] Zietak, M. , Kovatcheva‐Datchary, P. , Markiewicz, L. H. , Ståhlman, M. , & Kozak, L. P. (2016). Altered microbiota contributes to reduced diet‐induced obesity upon cold exposure. Cell Metabolism, 23(6), 1216–1223. 27304513 10.1016/j.cmet.2016.05.001 PMC4911343

[phy270838-bib-0174] Zuo, J. , Zhao, D. , Yu, N. , Fang, X. , Mu, Q. , Ma, Y. , Mo, F. , Wu, R. , Ma, R. , Wang, L. , Zhu, R. , Liu, H. , Zhang, D. , & Gao, S. (2017). Cinnamaldehyde ameliorates diet‐induced obesity in mice by inducing Browning of white adipose tissue. Cellular Physiology and Biochemistry, 42(4), 1514–1525. 28719892 10.1159/000479268

