# Supplementary material for: Cold exposure and metabolic health: Therapeutic potential for obesity, diabetes, and beyond
Source: Physiol Rep. 2026 Apr 7;14(7):e70838. doi: 10.14814/phy2.70838 (PMC13058241; doi:10.14814/phy2.70838)
Supplement: Supplementary file 1 — Table S1. [file PHY2-14-e70838-s001.doc]

Supplementary Table 1

| Reference year | Model | Food | Cold mode | Specific methods | Outcome | | | |
| --- | --- | --- | --- | --- | --- | --- | --- | --- |
|  |  |  |  |  | Food intake | BW | EE | BAT mass |
| Harri, M.,1984 | Rats | CD | Constant | 5±1 ℃ for at least 6 weeks | ↑ | ↓ | NA | ↑ |
| Yahata, T.,1989 | Rats | CD | Constant | 5 ℃ for 4 weeks | ↑ | ↓ | NA | ↑ |
| Park, G.,2023 | Mice | CD | Constant | Temperature was dropped by 4 ℃ per week from 18 ℃ to 6 ℃ for 4 weeks | ↑ | ↓ | NA | NA |
| Vallerand, A.L.,1986 | Rats | CD | Constant | 4℃ for 10 d | ↑ | ↓ | ↑ | ↑ |
| Yahata, T.,1989 | Rats | CD | Intermittent | 5 ℃ (6 h/d) for 4 weeks | ↑ | ↓ | NA | ↑ |
| Wang, T.Y.,2015 | Mice | CD | Intermittent | 4 ℃ (2 h/d; 5d/wks.) for 14 weeks | NA | ↔ | NA | ↔ |
| McKie, G.L.,2022 | Mice | CD | Intermittent | 29 ℃ for 6 weeks and then 4 ℃ (1 h/d;5 d/wks.) for 4 weeks | ↑ | ↔ | NA | ↑ |
| Harri, M.,1984 | Rats | CD | Intermittent | -20 ℃ (5 d/wks.) for 6 weeks Initial exposure time-10 min; Time extended by 3 min daily until the duration of the daily exposure time had reached 60 min | ↔ | ↔ | NA | ↑ |
| Aldiss, P.,2022 | Rats | HFD | Constant | 28℃ 3-12weks old and then 20℃ for 4 weeks | ↑ | ↑ | ↔ | ↑ |
| Vallerand, A.L.,1986 | Rats | HFD | Constant | 4℃ for 10 d | ↑ | ↓ | ↑ | ↑ |
| Ravussin, Y.,2014 | Mice | HFD | Intermittent | 4 ℃ for 0, 1,or 4 hours (Cohort 1) or 0, 4, or 8 hours (Cohort 2); three times a week for 10 weeks | ↑ | ↔ | ↔ | ↔ |
| McKie, G.L.,2022 | Mice | HFD | Intermittent | 29 ℃ for 6 weeks and then 4 ℃ (1 h/d;5 d/wks.) for 4 weeks | ↑ | ↑ | NA | ↑ |
| Smith, O.L.,1982 | Streptozotocin-induced diabetic rats | CD | Constant | 4℃ 24h | ↑ | ↔ | glucose level↓(mild diabetes) |  |
|  |  |  |  |  |  |  | glucose level↔(severe diabetes) |  |
| Li, F.X.,2024 | HFD+STZ induced T2DM  mice | HFD (9wks) CD (5wks) | Constant | 4-8℃ for 14 weeks | NA | ↔ | blood glucose↓  insulin sensitivity↑ |  |
| Sugimoto, S.,2022 | Mice | HFD | Constant | 5℃ for 2-7d | Systemic, BAT and liver inflammation↓ | BW ↓ | fasting glucose↓ | glucose tolerance↑ |
| Reynés, B.,2017 | Ferret | CD | Constant | 4℃ for 1weeks | Immune response-related pathways in aortic PVAT↓ |  |  |  |
| Castro, P.,2022 | Antigen-induced arthritis mice | CD | Intermittent | 20-minute ice-cold bag treatment every 2 hours, 2 sessions | Joint inflammation↓ |  |  |  |
| Guillot, X.,2017 | Rheumatoid arthritis (RA) rats | CD | Intermittent | Local cryotherapy (rats were treated by being placed into cages lined with ice pops for 30 mins , twice a day for 14 days) | Arthritis-related inflammation↓ |  |  |  |

CD：chow diet; HFD: high-fat diet; NA: not available
